# Unraveling the mechanism of [4Fe‐4S] cluster assembly on the N‐terminal cluster binding site of NUBP1

**DOI:** 10.1002/pro.4625

**Published:** 2023-05-01

**Authors:** Beatrice Bargagna, Sara Matteucci, Simone Ciofi‐Baffoni, Francesca Camponeschi, Lucia Banci

**Affiliations:** ^1^ Magnetic Resonance Center CERM, University of Florence, Via L. Sacconi 6, 50019 Sesto Fiorentino Florence Italy; ^2^ Consorzio Interuniversitario Risonanze Magnetiche di Metalloproteine (CIRMMP), Via L. Sacconi 6, 50019 Sesto Fiorentino Florence Italy; ^3^ Department of Chemistry University of Florence, Via della Lastruccia 3, 50019 Sesto Fiorentino Florence Italy

**Keywords:** anamorsin, electron transfer, GLRX3, iron–sulfur protein biogenesis, NUBP1

## Abstract

[4Fe‐4S]^2+^ cluster assembly in human cytosol requires both a [2Fe‐2S] cluster chaperone being able to donate two [2Fe‐2S]^2+^ clusters and an electron donor providing two electrons to reductively couple the two [2Fe‐2S]^2+^ clusters into a [4Fe‐4S]^2+^ cluster. The mechanism through which the cytosolic [4Fe‐4S]^2+^ cluster assembly works is still not defined. Here, we show that a hetero‐tetrameric complex formed by two molecules of cluster‐reduced [2Fe‐2S]^+^_2_‐anamorsin and one molecule of dimeric cluster‐oxidized [2Fe‐2S]^2+^_2_‐GLRX3_2_ orchestrates the assembly of a [4Fe‐4S]^2+^ cluster on the N‐terminal cluster binding site of the cytosolic protein NUBP1. We demonstrate that the hetero‐tetrameric complex is able to synergically provide two [2Fe‐2S]^2+^ clusters from GLRX3 and two electrons from anamorsin for the assembly of the [4Fe‐4S]^2+^ cluster on the N‐terminal cluster binding site of NUBP1. We also showed that only one of the two [2Fe‐2S] clusters bound to anamorsin, that is, that bound to the CX_8_CX_2_CXC motif, provides the electrons required to form the [4Fe‐4S]^2+^ cluster. Our study contributes to the molecular understanding of the mechanism of [4Fe‐4S] protein biogenesis in the cytosol.

## 1 INTRODUCTION

In the cytosol of eukaryotes, the maturation of [4Fe‐4S] proteins is carried out by a cytosolic iron–sulfur (Fe–S) cluster assembly (CIA) machinery (Ciofi‐Baffoni et al., 2018; Netz et al., 2014). An early proposed component of the CIA machinery is the cytosolic monothiol glutaredoxin GLRX3 (Braymer et al., 2021), a protein that dimerizes upon binding of two bridged [2Fe‐2S]^2+^ clusters ([2Fe‐2S]^2+^_2_‐GLRX3_2_, hereafter) (Camponeschi et al., 2020; Haunhorst et al., 2010). Among the proposed functions of GLRX3, there is its action as a [2Fe‐2S] cluster chaperone by donating its two [2Fe‐2S]^2+^ clusters to various partner proteins (Banci, Camponeschi, et al., 2015; Banci, Ciofi‐Baffoni, et al., 2015; Frey et al., 2016). One of these is NUBP1 (Camponeschi et al., 2020), another component of the CIA machinery, acting as a scaffold for the assembly of [4Fe‐4S] clusters together with its homologous NUBP2 (Stehling et al., 2008; Stehling et al., 2018). NUBP1 and NUBP2 belong to the deviant Walker A family NTPases (Grossman et al., 2019), and are both required for the CIA machinery (Stehling et al., 2008; Stehling et al., 2018). The two proteins form homo‐ and heterodimeric complexes, bridging a [4Fe‐4S] cluster bound to a CxxC motif close to the C‐termini of both proteins (Netz et al., 2012; Stehling et al., 2018). NUBP1 differs from NUBP2 as it harbors an N‐terminal conserved CX_13_CX_2_CX_5_C motif not present in NUBP2, which tightly binds a further [4Fe‐4S]^2+^ cluster (Camponeschi et al., 2020; Netz et al., 2012; Stehling et al., 2008). This N‐terminal iron–sulfur cluster is required for the CIA processes (Netz et al., 2012), but its role is not well understood. In the current working model, the N‐terminal iron–sulfur cluster remains stably bound to NUBP1 during the formation of the C‐terminal [4Fe‐4S]^2+^ cluster which is transiently bridged on the heterodimeric NUBP1‐NUBP2 complex as it is transferred along the CIA machinery pathway. Thus, at variance with the C‐terminal [4Fe‐4S]^2+^ cluster, the N‐terminal Fe–S cluster binding motif of NUBP1 behaves like a final acceptor of a [4Fe‐4S] cluster. The maturation of this [4Fe‐4S] cluster in yeast NUBP1 has been shown to be independent of the proteins acting later in the CIA machinery (Netz et al., 2007), thus differing markedly from the maturation of [4Fe‐4S] clusters into the majority of cytosolic target proteins that depends on the other components of the CIA machinery (Braymer et al., 2021; Ciofi‐Baffoni et al., 2018).

We recently investigated the mechanism for the formation of the N‐terminal [4Fe‐4S] cluster of NUBP1 in which two [2Fe‐2S]^2+^ clusters, donated by [2Fe‐2S]^2+^_2_‐GLRX3_2_ homodimer, are reductively coupled to form a [4Fe‐4S]^2+^ cluster, thanks to the electrons donated in vitro by glutathione (GSH) (Camponeschi et al., 2020). However, a possible physiological electron donor to form the [4Fe‐4S]^2+^ cluster in vivo at the N‐terminal site of NUBP1 is anamorsin, which is a partner protein of GLRX3 (Saito et al., 2011), and which receives electrons from another CIA component, the NADPH‐dependent diflavin reductase NDOR1 (Banci, Bertini, et al., 2013; Banci, Ciofi‐Baffoni, et al., 2013; Netz et al., 2010). This role of anamorsin is supported by the finding that the yeast homolog of anamorsin, that is, Dre2, is required for the Fe–S cluster assembly on Nbp35, but not on Cfd1, the yeast protein homologs of NUBP1 and NUBP2, respectively (Netz et al., 2010). As further support of anamorsin being the electron donor required to assemble the N‐terminal [4Fe‐4S]^2+^ cluster of NUBP1, it has been observed that, in *Arabidopsis thaliana*, the anamorsin homolog Dre2 specifically interacts with the NUBP1 homolog, NBP35 (Bastow et al., 2017). On the other hand, a direct experimental evidence of anamorsin acting as electron donor to assemble the [4Fe‐4S] cluster at the N‐terminal site of NUBP1 is lacking.

Anamorsin is an iron–sulfur cluster binding protein, structurally composed of a N‐terminal S‐adenosyl methionine methyl transferase‐like domain connected via a flexible linker to a largely unstructured C‐terminal cytokine‐induced apoptosis inhibitor 1 (CIAPIN1) domain (Banci et al., 2011; Soler et al., 2012; Song et al., 2014). The latter contains two conserved cysteine‐rich motifs (a CX_8_CX_2_CXC motif [M1, hereafter], followed by a CX_2_CX_7_CX_2_C motif [M2, hereafter]), each binding a [2Fe‐2S] cluster (Banci et al., 2011; Banci, Ciofi‐Baffoni, et al., 2013; Matteucci et al., 2021). The [2Fe‐2S] cluster bound at the M1‐motif is reduced by the diflavin reductase NDOR1 and the same reaction occurs between the yeast homolog proteins Dre2 and Tah18 (Banci, Ciofi‐Baffoni, et al., 2013; Netz et al., 2010). Conversely, the M2‐motif has been found to bind different types of clusters in different organisms, that is, a [2Fe‐2S] cluster in humans (Banci, Ciofi‐Baffoni, et al., 2013; Matteucci et al., 2021) and a [4Fe‐4S] cluster in yeast (Netz et al., 2016; Zhang et al., 2008; Zhang et al., 2017), and its function is still unknown in any organism (Netz et al., 2016; Zhang et al., 2017). The M2‐bound cluster is, however, not able to receive electrons from the diflavin reductase NDOR1 neither in humans nor in yeast (Banci, Ciofi‐Baffoni, et al., 2013; Netz et al., 2010), indicating that this Fe–S cluster does not have a redox function in the electron transfer process.

In this work, we characterized the mechanism for the assembly of the [4Fe‐4S]^2+^ cluster at the N‐terminal site of NUBP1, showing that GLRX3 and anamorsin are able to successfully perform it. We found that a hetero‐tetrameric complex, composed of homo‐dimeric [2Fe‐2S]^2+^_2_‐GLRX3_2_ and of two molecules of cluster‐reduced [2Fe‐2S]^+^_2_‐anamorsin, is able to provide two [2Fe‐2S]^2+^ clusters, from GLRX3, and two electrons, from anamorsin, thus assembling a [4Fe‐4S]^2+^ cluster on the N‐terminal site of NUBP1. We also found that only the [2Fe‐2S] cluster bound to the M1‐motif of [2Fe‐2S]_2_‐anamorsin, and not that bound to the M2‐motif, is the electron donor responsible of the reductive coupling of the two [2Fe‐2S]^2+^ clusters to form the [4Fe‐4S]^2+^ cluster on the N‐terminal site of NUBP1.

## 2 RESULTS

### 2.1 Formation of a [4Fe‐4S]^2+^ cluster on the N‐terminal site of NUBP1 driven by GLRX3 and anamorsin

**FIGURE 1:**
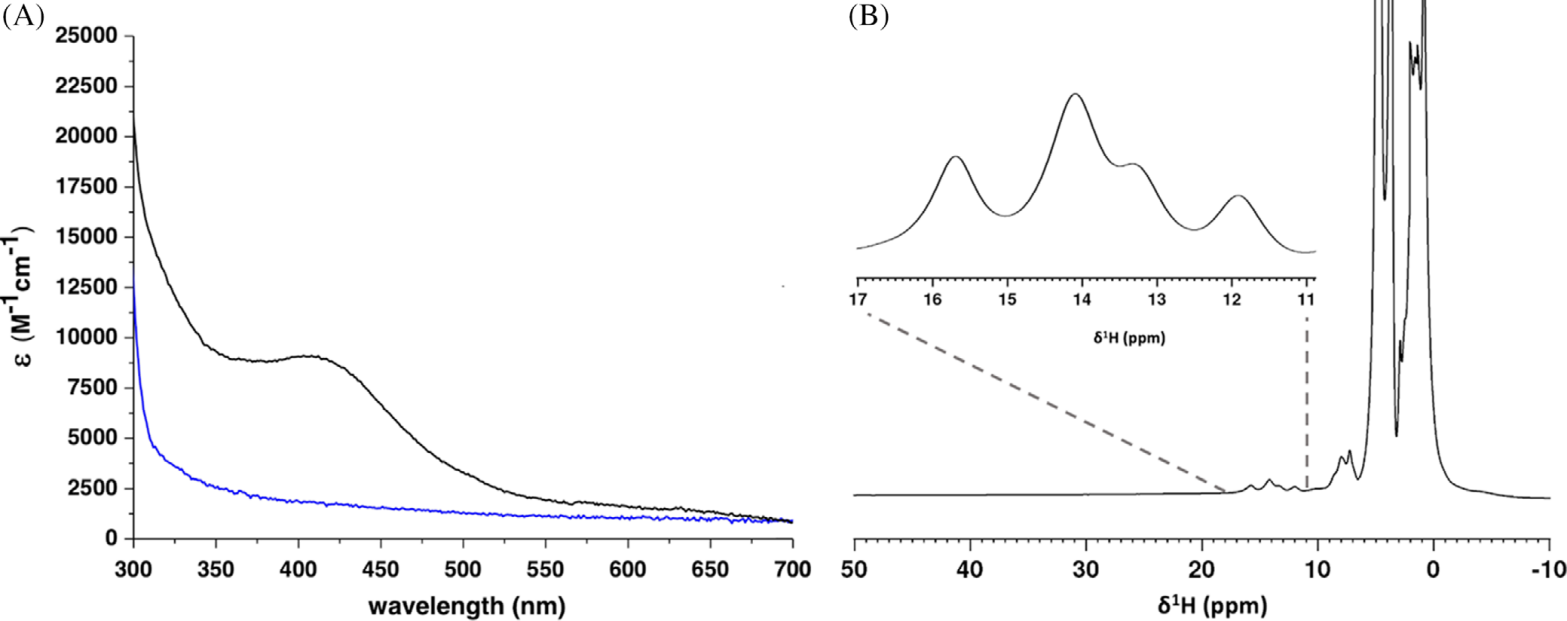
[2Fe‐2S]^2+^_2_‐GLRX3_2_/[2Fe‐2S]^+^_2_‐anamorsin mixture promotes the assembly of a [4Fe‐4S]^2+^ cluster at the N‐terminal site of His_6_‐tagged NUBP1. (A) UV–vis spectra of His_6_‐tagged NUBP1 before (blue line) and after (black line) the incubation and isolation from [2Fe‐2S]^2+^_2_‐GLRX3_2_ and [2Fe‐2S]^+^_2_‐anamorsin; (B) paramagnetic 1D ^1^H NMR spectrum of His_6_‐tagged NUBP1 after incubation and isolation from [2Fe‐2S]^2+^_2_‐GLRX3_2_ and [2Fe‐2S]^+^_2_‐anamorsin.

His_6_‐tagged apo NUBP1 was mixed, under anaerobic conditions, with chemically reconstituted, untagged [2Fe‐2S]^2+^_2_‐GLRX3_2_ and with untagged [2Fe‐2S]^+^_2_‐anamorsin with both clusters in the reduced state (see Materials and Methods for details). After ~1 hour, His_6_‐tagged NUBP1 was isolated from GLRX3 and anamorsin, using Ni^2+^‐affinity chromatography. The protein content of the collected fractions was assessed by polyacrylamide gel electrophoresis (SDS‐PAGE) (Figure S1), and the fraction containing isolated His_6_‐tagged NUBP1 protein was characterized by UV–visible (UV–vis) and NMR spectroscopies, and by performing acid‐labile sulfide and iron quantification. The UV–vis spectrum of isolated His_6_‐tagged NUBP1 is characteristic of a [4Fe‐4S]^2+^‐bound protein, with the presence of a broad absorbance band at ~410 nm (Figure 1A). The paramagnetic 1D ^1^H NMR spectrum of the isolated His_6_‐tagged NUBP1 showed four hyperfine shifted signals in the 18–11 ppm spectral region (Figure 1B), whose chemical shift values and temperature dependence (Figure S2) are typical of βCH_2_ of cysteines bound to an oxidized [4Fe‐4S]^2+^ cluster (Banci et al., 2018; Bertini et al., 1994). These signals are well superimposable with those previously observed for the four N‐terminal cysteines of NUBP1 bound to an oxidized [4Fe‐4S]^2+^ cluster (Camponeschi et al., 2020). Overall, these data indicate that the [2Fe‐2S]^2+^_2_‐GLRX3_2_/[2Fe‐2S]^+^_2_‐anamorsin mixture is able to form a [4Fe‐4S]^2+^ cluster on the N‐terminal site of His_6_‐tagged NUBP1. Acid‐labile sulfide and iron analysis showed the presence of ~0.6 [4Fe‐4S] clusters per His_6_‐tagged NUBP1 molecule (Table 1).

**TABLE 1:** Iron and acid‐labile sulfide quantification of His_6_‐tagged NUBP1 after cluster transfer/assembly reaction.

Sample	Fe^a^	S^a^	[2Fe‐2S]	[4Fe‐4S]
His_6_‐tagged NUBP1 isolated from [2Fe‐2S]^2+^_2_‐GLRX3_2_ and [2Fe‐2S]^+^_2_‐WT‐anamorsin mixture	2.3 ± 0.1	2.3 ± 0.1	‐	0.6 ± 0.1
His_6_‐tagged NUBP1_NT_ isolated from [2Fe‐2S]^2+^_2_‐GLRX3_2_ and [2Fe‐2S]^+^_2_‐WT‐anamorsin mixture	2.7 ± 0.1	2.8 ± 0.1	‐	0.7 ± 0.1
His_6_‐tagged NUBP1_CT_ isolated from [2Fe‐2S]^2+^_2_‐GLRX3_2_ and [2Fe‐2S]^+^_2_‐WT‐anamorsin mixture	0.3 ± 0.1	0.2 ± 0.1	‐	‐
His_6_‐tagged NUBP1 isolated from [2Fe‐2S]^2+^_2_‐GLRX3_2_ and [2Fe‐2S]^+^‐M1‐anamorsin mixture	2.6 ± 0.1	2.9 ± 0.1	‐	0.7 ± 0.1
His_6_‐tagged NUBP1 isolated from [2Fe‐2S]^2+^_2_‐GLRX3_2_ and [2Fe‐2S]^+^‐M2‐anamorsin mixture	1.1 ± 0.1	1.2 ± 0.1	0.6 ± 0.1	‐

^a^ Fe and acid‐labile S measurements are reported as mol Fe or S per mol of His_6_‐tagged NUBP1 molecule. Data are the average of three independent samples.

To further support that a [4Fe‐4S] cluster is assembled at the N‐terminal site of NUBP1 and to rule out that no [4Fe‐4S] cluster is assembled at the C‐terminal site of NUBP1, we mixed under anaerobic conditions untagged [2Fe‐2S]^2+^_2_‐GLRX3_2_ and untagged, cluster‐reduced [2Fe‐2S]^+^_2_‐anamorsin with a His_6_‐tagged variant of NUBP1 where the two cysteines in the CPXC motif where mutated into alanines, thus containing only the N‐terminal cluster binding motif (NUBP1_NT_, hereafter) or with a His_6_‐tagged construct of NUBP1 where the first 37 N‐terminal residues were deleted, thus containing only the C‐terminal CPXC cluster binding motif (NUBP1_CT_, hereafter). The UV–visible spectrum of isolated His_6_‐tagged NUBP1_NT_ showed the presence of a broad absorption band at ~410 nm (Figure S3A), indicating the binding of a [4Fe‐4S]^2+^ cluster to the protein. Acid‐labile sulfide and iron analysis showed the presence of ~0.7 [4Fe‐4S] clusters per NUBP1_NT_ molecule (Table 1), which is comparable with what observed for full‐length wild‐type NUBP1. Conversely, the UV–vis spectrum of isolated His_6_‐tagged NUBP1_CT_ did not show a significant absorption band arising from a bound Fe–S cluster (Figure S3B). Overall, these data indicate that the [2Fe‐2S]^2+^_2_‐GLRX3_2_/[2Fe‐2S]^+^_2_‐anamorsin mixture is able to assemble a [4Fe‐4S]^2+^ cluster at the N‐terminal cluster binding site of NUBP1, but not at the C‐terminal site.

**FIGURE 2:**
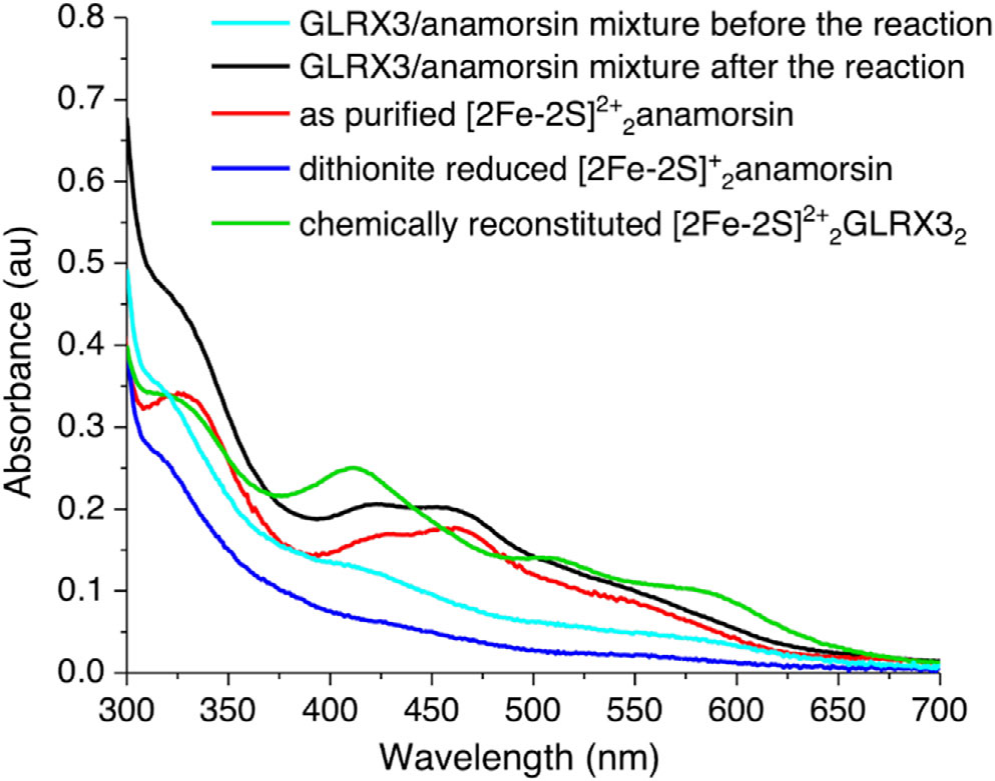
[2Fe‐2S]^+^_2_‐anamorsin provides electrons and [2Fe‐2S]^2+^_2_‐GLRX3_2_ provides the [2Fe‐2S]^2+^ clusters for the assembly of a [4Fe‐4S]^2+^ cluster at the N‐terminal site of His_6_‐tagged NUBP1. UV–vis spectra of [2Fe‐2S]^2+^_2_‐GLRX3_2_/[2Fe‐2S]^+^_2_‐anamorsin mixture before (cyan line) and after (black line) the incubation and separation from His_6_‐tagged NUBP1. The spectra of isolated [2Fe‐2S]^2+^_2_‐GLRX3_2_ (green line), [2Fe‐2S]^+^_2_‐anamorsin (blue line) and [2Fe‐2S]^2+^_2_‐anamorsin (red line) are reported for reference.

UV–vis spectroscopy was also performed on the [2Fe‐2S]^2+^_2_‐GLRX3_2_/[2Fe‐2S]^+^_2_‐anamorsin mixture before its incubation with His_6_‐tagged apo NUBP1 and then after its separation from the latter. The spectrum of the GLRX3/anamorsin mixture before incubation with NUBP1 shows faint bands at 330, 420, 510, and 580 nm (Figure 2, cyan line), which are typical of cluster‐reduced [2Fe‐2S]^+^_2_‐anamorsin (Figure 2, blue line) and of oxidized [2Fe‐2S]^2+^_2_‐GLRX3_2_ (Figure 2, green line). Upon incubation and isolation from His_6_‐tagged NUBP1, the spectrum of the GLRX3/anamorsin mixture changed significantly, showing intense absorption bands at 320, 430, and 460 nm (Figure 2A, black line), that are characteristic of the oxidized [2Fe‐2S]^2+^_2_‐bound form of anamorsin (Figure 2A, red line). These data clearly showed that anamorsin gets oxidized after incubation with His_6_‐tagged NUBP1. Moreover, the spectrum of the GLRX3/anamorsin mixture after incubation with His_6_‐tagged apo NUBP1 does not show the bands at 510 and 580 nm, typical of the [2Fe‐2S]^2+^ clusters bound to GLRX3, indicating that GLRX3 has no bound cluster anymore and thus that it transfers its clusters to NUBP1.

To better define the specific roles played by GLRX3 and anamorsin in the [4Fe‐4S] cluster assembly process, His_6_‐tagged apo NUBP1 was incubated with cluster‐reduced, untagged [2Fe‐2S]^+^_2_‐anamorsin in the absence of [2Fe‐2S]^2+^_2_‐GLRX3_2_. No bands were observed in the UV–vis spectrum of isolated His_6_‐tagged NUBP1, indicating that no cluster transfer occurs from anamorsin to NUBP1 in the absence of GLRX3 (Figure S4). His_6_‐tagged apo NUBP1 was also mixed with chemically reconstituted, untagged [2Fe‐2S]^2+^_2_‐GLRX3_2_ in the absence of anamorsin. The UV–vis spectrum of isolated His_6_‐tagged NUBP1 showed absorption bands at 330 and 420 nm, with the latter having a shoulder at ~460 nm and a broad unresolved band centered at ~580 nm (Figure S5A), which are characteristic of a [2Fe‐2S]^2+^ cluster bound to NUBP1. These results indicate that [2Fe‐2S]^2+^_2_‐GLRX3_2_ can transfer its [2Fe‐2S]^2+^ clusters cargo to NUBP1 in the absence of anamorsin and support that cluster reduced, [2Fe‐2S]^+^_2_‐anamorsin is required for the assembly of the [4Fe‐4S]^2+^ cluster on NUBP1.

To gain more insight on the role played by [2Fe‐2S]^+^_2_‐anamorsin, His_6_‐tagged apo NUBP1 was mixed with chemically reconstituted, untagged [2Fe‐2S]^2+^_2_‐GLRX3_2_ and untagged, cluster‐oxidized [2Fe‐2S]^2+^_2_‐anamorsin. The UV–vis spectrum of isolated NUBP1 showed absorption bands at 420 nm, 460 nm, and 580 nm (Figure S5B), that are indicative of the binding of a [2Fe‐2S]^2+^ cluster. These data clearly showed that electron transfer from the GLRX3/anamorsin mixture to NUBP1 does not occur when both clusters of anamorsin are in their oxidized state, thus supporting the electron transfer function of [2Fe‐2S]^+^_2_‐anamorsin in the [4Fe‐4S] cluster assembly process.

Overall, these data indicate that reduced [2Fe‐2S]^+^_2_‐anamorsin is able to promote [4Fe‐4S]^2+^ assembly on the N‐terminal site of NUBP1, by providing the electrons required to reductively couple two [2Fe‐2S]^2+^ clusters donated by [2Fe‐2S]^2+^_2_‐GLRX3_2_.

### 2.2 Electrons for the assembly of the [4Fe‐4S]^2+^ cluster at the N‐terminal site of NUBP1 are provided by the M1‐bound [2Fe‐2S] cluster of anamorsin

Since it was not possible to selectively reduce either the M1‐bound or the M2‐bound [2Fe‐2S] cluster of anamorsin, both [2Fe‐2S] clusters were reduced in the previous set of experiments, and therefore both clusters might be involved in the electron transfer process. Thus, we investigated the role of the M1‐ and M2‐motifs in the electron transfer by performing a new set of experiments, analogous to those performed with wild‐type anamorsin (WT‐anamorsin, hereafter), but in the presence of: (i) a construct of anamorsin lacking the last 49 C‐terminal residues, and therefore containing only the M1‐motif (M1‐anamorsin, hereafter), and (ii) a mutant containing only the M2‐motif, as the four cysteines of the M1‐motif were mutated into alanines (M2‐anamorsin, hereafter). Each of the cluster‐binding sites in the anamorsin variants were previously shown by us to maintain the same cluster coordination and electronic properties of the wild‐type protein (Banci, Ciofi‐Baffoni, et al., 2013; Matteucci et al., 2021), indicating that the two cys‐rich motifs can independently bind a [2Fe‐2S] cluster, and thus that the presence of one cluster does not affect the redox properties of the other cluster in the molecule.

**FIGURE 3:**
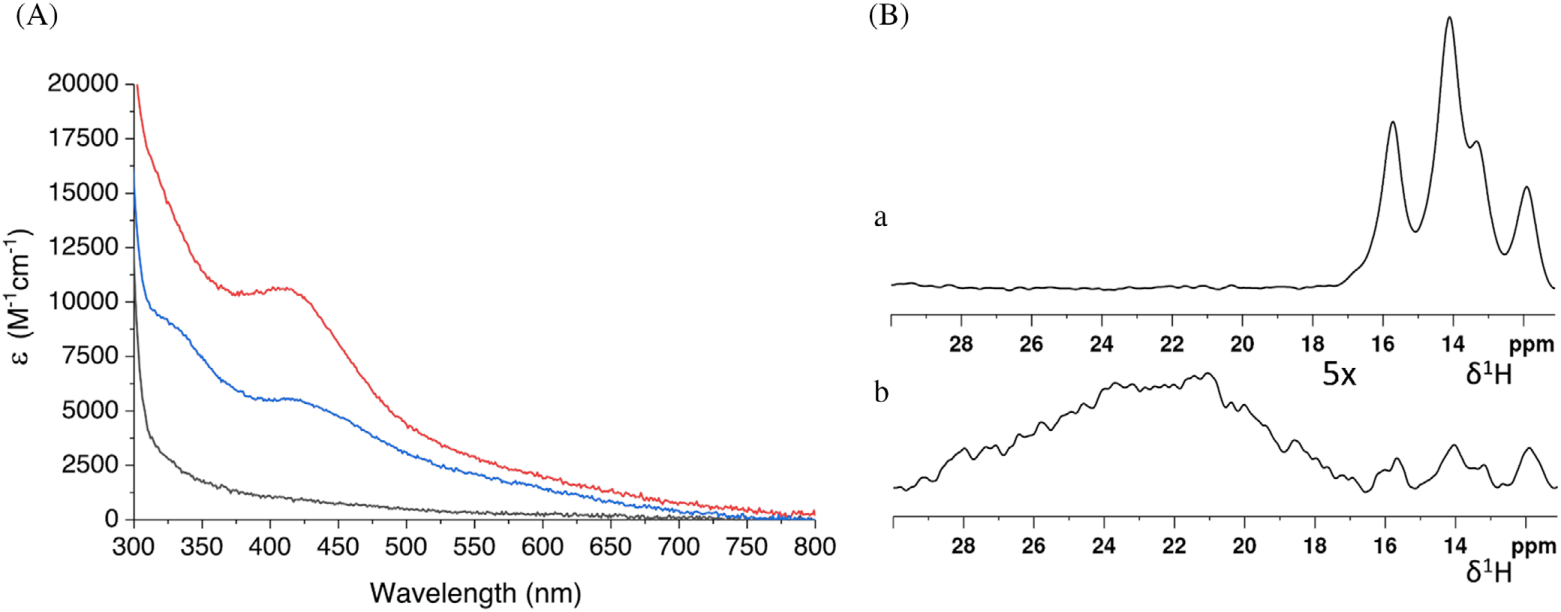
The M1‐bound [2Fe‐2S]^+^ cluster of anamorsin provides electrons for the assembly of the [4Fe‐4S]^2+^ cluster at the N‐terminal site of NUBP1. (A) UV–vis spectra of His_6_‐tagged NUBP1 before (black line) and after the incubation and isolation from [2Fe‐2S]^2+^_2_‐GLRX3_2_ and M1‐ (red line) and M2‐anamorsin (blue line); (B) paramagnetic 1D ^1^H NMR spectra of His_6_‐tagged NUBP1 after incubation and isolation from [2Fe‐2S]^2+^_2_‐GLRX3_2_ and [a] M1‐ and [b] M2‐anamorsin (5 times magnification).

After incubation of His_6_‐tagged NUBP1 with the [2Fe‐2S]^2+^_2_‐GLRX3_2_/[2Fe‐2S]^+^‐M1‐anamorsin mixture followed by its isolation, His_6_‐tagged NUBP1 showed a broad absorbance band at ~410 nm in the UV–vis spectra, characteristic of the oxidized [4Fe‐4S]^2+^ cluster‐bound form of NUBP1 (Camponeschi et al., 2020) (Figure 3A, red line). Accordingly, the UV–vis CD spectrum of His_6_‐tagged NUBP1 was essentially featureless (Figure S6), in agreement with the binding of an oxidized [4Fe‐4S]^2+^ cluster. Moreover, the four sharp hyperfine shifted signals, characteristic of the oxidized [4Fe‐4S]^2+^ cluster bound to the N‐terminal site of NUBP1 (Camponeschi et al., 2020), were observed in the paramagnetic 1D ^1^H NMR spectrum of His_6_‐tagged NUBP1 isolated after the reaction (Figure 3B, a). Acid‐labile sulfide and iron analysis showed the presence of ~0.7 [4Fe‐4S] clusters per NUBP1 molecule (Table 1), which is comparable with what observed with WT‐anamorsin. Therefore, these data reproduce those observed for WT‐anamorsin, indicating that the M1‐cluster provides electrons to assemble the [4Fe‐4S]^2+^ cluster on the N‐terminal site of NUBP1.

On the contrary, when [2Fe‐2S]^+^‐M2‐anamorsin was used in the reaction, the UV–vis spectrum of the isolated His_6_‐tagged NUBP1 showed absorption bands centered at 330 and at 420 nm, the latter with a shoulder at ~460 nm (Figure 3A, blue line). UV–vis CD spectrum of the isolated His_6_‐tagged NUBP1 showed positive bands centered at 327 and 476 nm, and negative bands centered at 436 and 530 nm (Figure S6). Both the UV–vis absorption and CD spectra are characteristic of a [2Fe‐2S]^2+^ cluster, thus indicating the transfer of a [2Fe‐2S]^2+^ cluster from GLRX3 to NUBP1, without the assembly of a significant amount of [4Fe‐4S]^2+^ cluster. The 1D ^1^H NMR spectrum of isolated His_6_‐tagged NUBP1 from the latter mixture is in full agreement with this result, showing indeed a broad, unresolved main signal in the 30–20 ppm region (Figure 3B, b), typical of βCH_2_ of cysteines bound to a [2Fe‐2S]^2+^ cluster (Banci et al., 1990; Banci et al., 2018), and very low intensity signals of βCH_2_ of cysteines bound to the N‐terminal [4Fe‐4S]^2+^ cluster in the 16–11 ppm region (Figure 3B, b). These findings indicate that the reduced, M2‐bound [2Fe‐2S] cluster of anamorsin does not provide the electrons required for the assembly of a [4Fe‐4S]^2+^ cluster on the N‐terminal site of NUBP1 and does not play a role in the [4Fe‐4S]^2+^ cluster assembly process.

To analyze the variations in the redox state of the two clusters bound to the M1 and M2 motifs of anamorsin in the NUBP1 [4Fe‐4S]^2+^ cluster assembly process, we applied Continuous Wave (CW) X‐band EPR and paramagnetic 1D ^1^H NMR spectroscopy to the [2Fe‐2S]^2+^_2_‐GLRX3_2_/[2Fe‐2S]^+^_2_‐WT‐anamorsin/NUBP1 reaction mixture.

**FIGURE 4:**
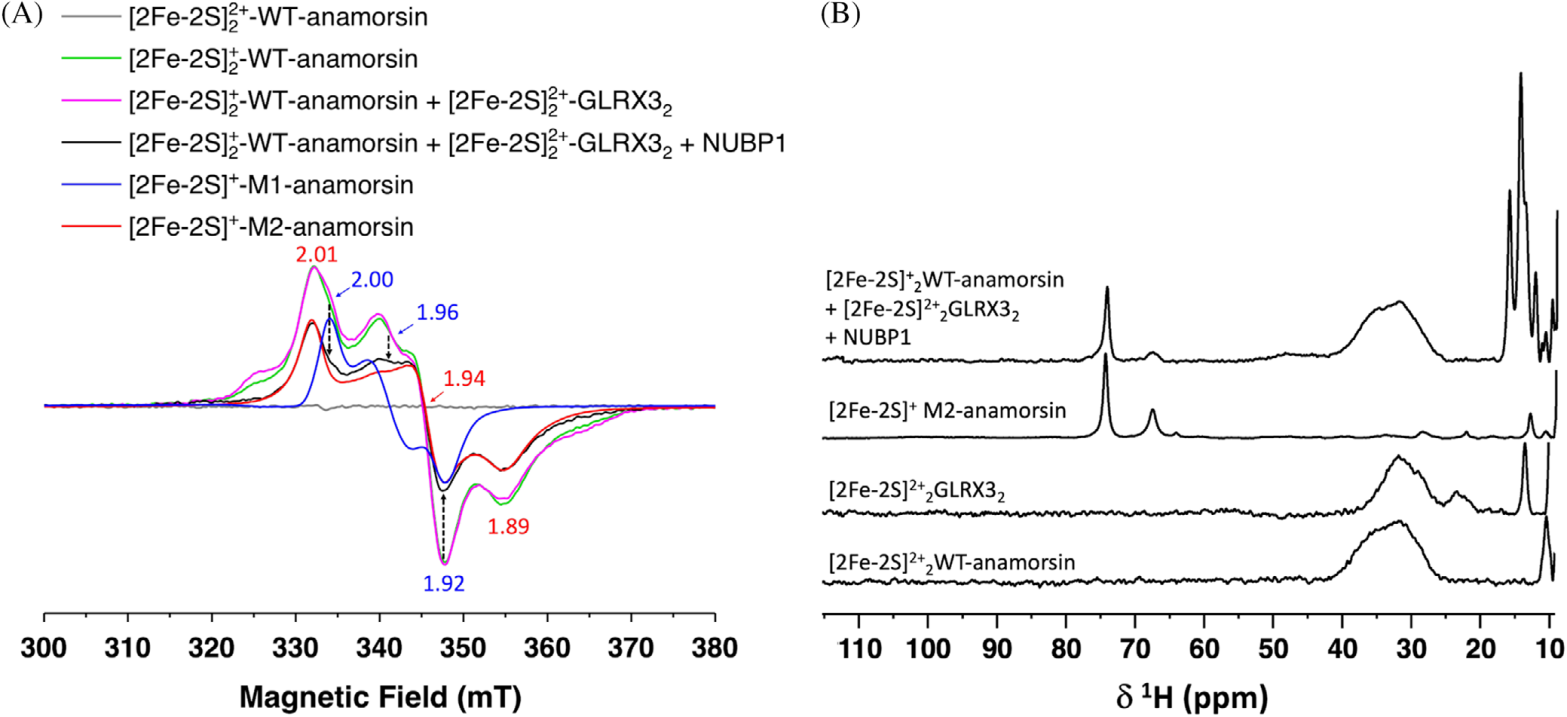
The M2‐bound [2Fe‐2S]^+^ cluster of anamorsin does not transfer electrons for the assembly of a [4Fe‐4S] cluster on NUBP1. (A) CW X‐band EPR spectra at 10 K, 1 mW of: cluster‐oxidized [2Fe‐2S]^2+^_2_‐WT‐anamorsin (gray line); dithionite reduced [2Fe‐2S]^+^_2_‐WT‐anamorsin (green line); a mixture of [2Fe‐2S]^2+^_2_‐GLRX3_2_ and dithionite reduced [2Fe‐2S]^+^_2_‐WT‐anamorsin (magenta line); a mixture of [2Fe‐2S]^2+^_2_‐GLRX3_2_, dithionite reduced [2Fe‐2S]^+^_2_‐WT‐anamorsin and NUBP1 (black line); dithionite reduced [2Fe‐2S]^+^‐M1‐anamorsin (blue line); dithionite reduced [2Fe‐2S]^+^‐M2‐anamorsin (red line). *g* values for reduced M1‐ and M2‐bound [2Fe‐2S]^+^ clusters are indicated in blue and red, respectively. The black, dotted arrows indicate the decrease in intensity of the EPR signal of the reduced M1‐bound [2Fe‐2S] cluster of [2Fe‐2S]^+^_2_‐WT‐anamorsin upon addition of His_6_‐tagged NUBP1 to the mixture of [2Fe‐2S]^2+^_2_‐GLRX3_2_ and dithionite reduced [2Fe‐2S]^+^_2_‐WT‐anamorsin. (B) From top to bottom: 1D ^1^H paramagnetic NMR spectra of a mixture of [2Fe‐2S]^2+^_2_‐GLRX3_2_, dithionite reduced [2Fe‐2S]^+^_2_‐WT‐anamorsin and apo NUBP1; isolated dithionite reduced [2Fe‐2S]^+^‐M2‐anamorsin; isolated [2Fe‐2S]^2+^_2_‐GLRX3_2_; isolated cluster‐oxidized [2Fe‐2S]^2+^_2_‐WT‐anamorsin. NMR spectra were acquired at 400 MHz, 298 K.

While both clusters of anamorsin are EPR‐silent in their [2Fe‐2S]^2+^ oxidized states, having a *S* = 0 ground spin state (Figure 4A, gray line), the *S* = 1/2 ground spin state of each of the two reduced M1‐ and M2‐bound [2Fe‐2S]^+^ clusters have distinct EPR signals (Banci, Ciofi‐Baffoni, et al., 2013; Matteucci et al., 2021), that allow to easily discriminate them. The EPR spectra of dithionite‐reduced [2Fe‐2S]^+^_2_‐WT‐anamorsin (Figure 4A, green line) and of the mixture composed of [2Fe‐2S]^2+^_2_‐GLRX3_2_ and dithionite‐reduced [2Fe‐2S]^+^_2_‐WT‐anamorsin before the incubation with apo NUBP1 (Figure 4A, magenta line), showed, at 10 K and 1 mW, EPR features arising from two distinct rhombic EPR signals, with principal g values of 2.00, 1.96, 1.92 and of 2.01, 1.94, 1.89, that were previously assigned to the two reduced [2Fe‐2S]^+^ clusters bound to the M1 and M2 motifs of WT‐anamorsin, respectively (Banci, Ciofi‐Baffoni, et al., 2013; Matteucci et al., 2021), and that were observed in the EPR spectra of the two isolated cluster‐reduced M1‐ and M2‐anamorsin constructs (Figure 4A, blue and red lines, respectively). Upon incubation with apo NUBP1, the signal originating from the reduced M1‐bound [2Fe‐2S]^+^ cluster significantly decreased in the spectrum of the mixture of [2Fe‐2S]^2+^_2_‐GLRX3_2_, dithionite‐reduced [2Fe‐2S]^+^_2_‐anamorsin and NUBP1 (Figure 4A, black line). Conversely, the EPR signal originating from the reduced M2‐bound [2Fe‐2S]^+^ cluster did not change in intensity, accounting for the majority of the EPR signal, as showed by the comparison with the EPR spectrum recorded on isolated dithionite‐reduced M2‐anamorsin (Figure 4A, red line).

Paramagnetic 1D ^1^H NMR spectrum acquired on the [2Fe‐2S]^2+^_2_‐GLRX3_2_, [2Fe‐2S]^+^_2_‐WT‐anamorsin and apo NUBP1 reaction mixture showed the presence of several sets of hyperfine shifted signals, originating from different paramagnetic species which are formed as a result of the [4Fe‐4S]^2+^ cluster assembly on the N‐terminal site of NUBP1 (Figure 4B). Specifically, four well resolved sharp signals arising from the [4Fe‐4S]^2+^‐cluster bound form of NUBP1 were observed in the 18–11 ppm region. In addition to these signals, the spectrum showed a broad, unresolved signal in the 40–25 ppm region, typical of βCH_2_ of cysteine residues bound to oxidized [2Fe‐2S]^2+^ clusters (Banci et al., 2018). This signal fully reproduces the shape of that observed for as purified, cluster‐oxidized WT‐anamorsin (Figure 4B; Banci et al., 2011). Two further signals are present in the ^1^H NMR spectrum of the reaction mixture at 76 and 68 ppm, whose chemical shift values and linewidths are typical of βCH_2_ of cysteines bound to a reduced [2Fe‐2S]^+^ cluster (Banci et al., 2018). These signals corresponds to those observed in the 1D ^1^H NMR spectrum of isolated, cluster‐reduced [2Fe‐2S]^+^‐M2‐anamorsin (Figure 4B), thus indicating that the [2Fe‐2S] cluster bound to the M2‐motif of anamorsin remains in a reduced redox state after the [4Fe‐4S]^2+^ cluster assembly on the N‐terminal site of NUBP1. Finally, the signal at 22 ppm which is a unique feature of [2Fe‐2S]^2+^_2_‐GLRX3_2_ as showed by the paramagnetic 1D ^1^H NMR spectrum of isolated [2Fe‐2S]^2+^_2_‐GLRX3_2_ (Figure 4B), is absent in the spectrum of the [2Fe‐2S]^2+^_2_‐GLRX3_2_, [2Fe‐2S]^+^_2_‐WT‐anamorsin and apo NUBP1 mixture, indicating that, in the latter, [2Fe‐2S]^2+^_2_‐GLRX3_2_ is not present anymore and thus confirming the transfer of the [2Fe‐2S]^2+^ clusters from GLRX3 to the N‐terminal site of NUBP1.

Overall, the EPR and paramagnetic 1D ^1^H NMR data showed that, upon the [4Fe‐4S]^2+^ cluster assembly on the N‐terminal site of NUBP1, anamorsin is partially oxidized, that is, with the M2‐motif still binding a reduced [2Fe‐2S]^+^ cluster, while the cluster bound to the M1‐motif gets oxidized, thus indicating that only the M1‐bound and not the M2‐bound [2Fe‐2S] cluster of anamorsin acts as an electron donor for the assembly of [4Fe‐4S]^2+^ cluster on the N‐terminal site of NUBP1.

### 2.3 [2Fe‐2S] _2_‐GLRX3_2_ /[2Fe‐2S]_2_‐anamorsin hetero‐tetrameric complex is the key intermediate driving the assembly of a [4Fe‐4S]^2+^ cluster on the N‐terminal site of NUBP1

To further characterize the mechanism of the GLRX3/anamorsin‐dependent maturation of the [4Fe‐4S]^2+^ cluster bound to the N‐terminal site of NUBP1, we investigated, by analytical SEC, the interaction between [2Fe‐2S]^2+^_2_‐GLRX3_2_ and full‐length [2Fe‐2S]_2_‐WT‐anamorsin (having either oxidized or reduced clusters). The analytical SEC of [2Fe‐2S]_2_‐WT‐anamorsin as well as that of the [2Fe‐2S]_2_‐GLRX3/[2Fe‐2S]_2_‐anamorsin protein mixture did not show different profiles when the [2Fe‐2S] clusters of anamorsin were reduced or oxidized. The redox state of the [2Fe‐2S] clusters of anamorsin is thus not reported hereafter.

**FIGURE 5:**
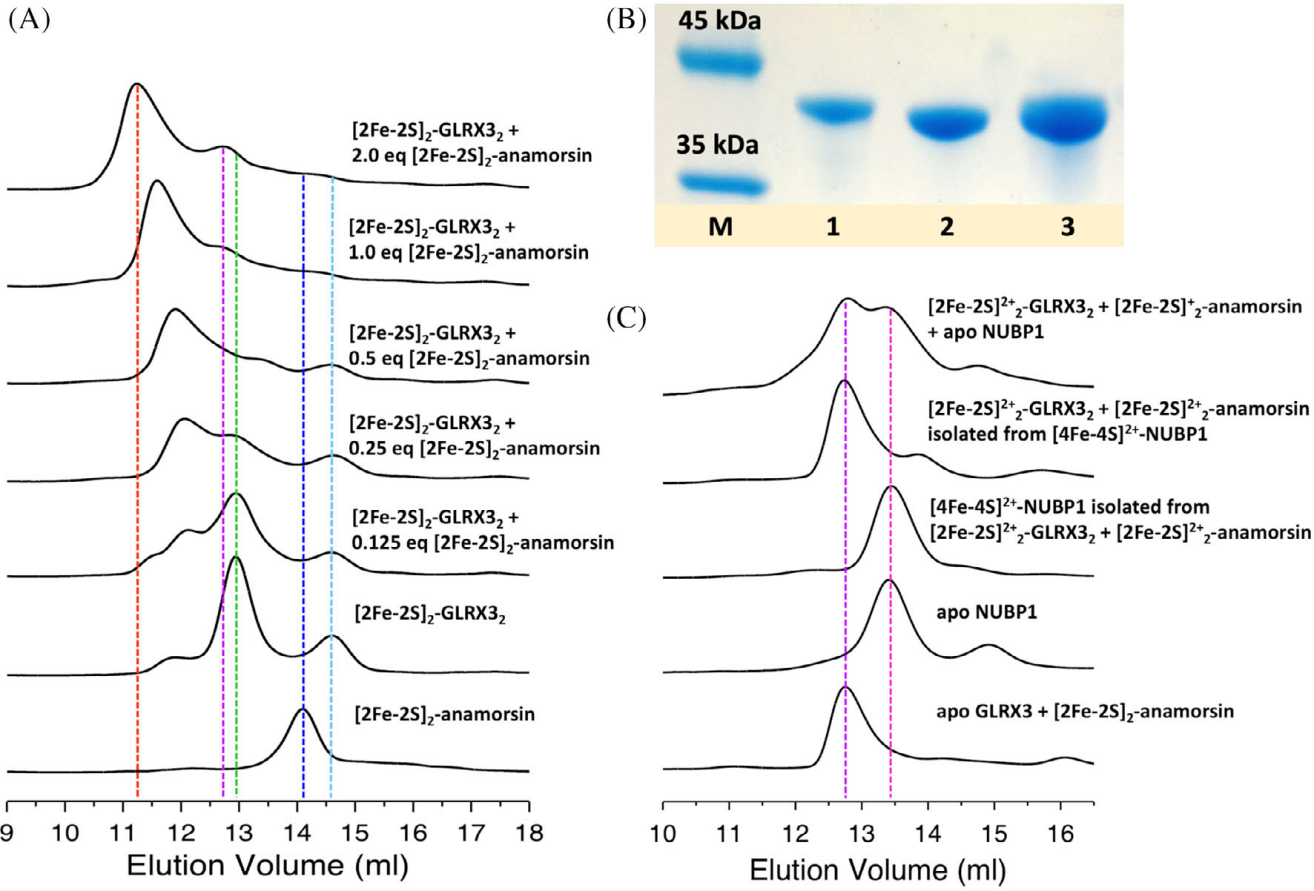
[2Fe‐2S]_2_‐GLRX3_2_ and [2Fe‐2S]_2_‐anamorsin form a hetero‐tetrameric complex that drives the assembly of the [4Fe‐4S]^2+^ cluster at the N‐terminal site of NUBP1. (A) Analytical SEC of [2Fe‐2S]_2_‐GLRX3_2_/[2Fe‐2S]_2_‐WT‐anamorsin mixtures composed as indicated in the figure. Vertical, dotted lines indicate the peaks corresponding to the main species: cyan = apo GLRX3; green = [2Fe‐2S]_2_‐GLRX3_2_; blue = [2Fe‐2S]^2+^_2_‐anamorsin; red = a 1:2 [2Fe‐2S]_2_‐GLRX3_2_:[2Fe‐2S]_2_‐anamorsin complex. (B) SDS‐PAGE analysis of the protein content of the SEC peak corresponding to the hetero‐tetrameric 1:2 [2Fe‐2S]_2_‐GLRX3_2_:[2Fe‐2S]_2_‐anamorsin complex in panel A. M = marker; lane 1 = isolated [2Fe‐2S]_2_‐anamorsin; lane 2 = isolated [2Fe‐2S]_2_‐GLRX3_2_; lane 3 = main peak of the top line in panel A, corresponding to the GLRX3/anamorsin hetero‐tetrameric complex. (C) Analytical SEC of the products of the cluster transfer/assembly reaction before and after separation by Ni^2+^‐affinity chromatography, of apo His_6_‐tagged NUBP1 before incubation and of the 1:1 apo GLRX3/[2Fe‐2S]_2_‐anamorsin heterodimeric complex. Vertical, dotted lines indicate the peaks corresponding to the main species: purple = apo GLRX3:[2Fe‐2S]_2_‐anamorsin 1:1 complex; magenta = His_6_‐tagged NUBP1 before and after the reaction.

[2Fe‐2S]^2+^_2_‐GLRX3_2_ elutes with two peaks (Figure 5A, green and cyan lines), corresponding to homodimeric [2Fe‐2S]^2+^_2_‐GLRX3_2_ (13.0 mL) and apo monomeric GLRX3 (14.5 mL), with apparent molecular masses MW_app_ = 132.5 kDa and MW_app_ = 62.4 kDa, respectively. [2Fe‐2S]_2_‐WT‐anamorsin elutes with a main peak at 14.09 mL (Figure 5A, blue line), with MW_app_ = 78.7 kDa. The apparent molecular masses obtained for both GLRX3 and WT‐anamorsin are higher than the theoretical ones (Table S1), in agreement with what previously reported by us (Banci, Camponeschi, et al., 2015). This behavior is in line with what expected for multidomain proteins, such as GLRX3 and anamorsin, which are also characterized by a dynamic multiconformational heterogeneity that gives rise to an overall larger hydrodynamic radius, resulting in an increase of the apparent molecular mass of the isolated proteins as well as of their protein–protein complexes (Table S1). Upon sub‐stoichiometric additions of [2Fe‐2S]_2_‐WT‐anamorsin to [2Fe‐2S]^2+^_2_‐GLRX3_2_, the peak at 13.0 mL progressively decreases in intensity, while the peak at 14.5 mL remains approximately unchanged. Concomitantly, a new peak with ~12 mL elution volume appears (Figure 5A). This peak shifts to lower volumes upon further [2Fe‐2S]_2_‐WT‐anamorsin additions. The lowest elution volume was reached upon addition of 2 eq. of [2Fe‐2S]_2_‐WT‐anamorsin, with a main peak eluting at 11.2 mL (Figure 5A, red line). The SDS‐PAGE protein content analysis of the peak eluting at 11.2 mL showed the presence of both GLRX3 and anamorsin (Figure 5B) and the apparent molecular mass of the new species (MW_app_ = 288.4 kDa) is very close to the MW_app_ expected for a hetero‐tetrameric complex between one [2Fe‐2S]^2+^_2_‐GLRX3_2_ dimer and two [2Fe‐2S]_2_‐WT‐anamorsin molecules (i.e., 289.8 kDa, calculated from the sum of the apparent molecular masses of [2Fe‐2S]^2+^_2_‐GLRX3_2_ and [2Fe‐2S]_2_‐WT‐anamorsin, Table S1). The elution volume shift of this peak from 12 to 11.2 mL can be interpreted as an equilibrium between the hetero‐tetrameric complex and a hetero‐trimeric complex formed by one [2Fe‐2S]^2+^_2_‐GLRX3_2_ dimer and one molecule of [2Fe‐2S]_2_‐WT‐anamorsin, which are in fast exchange in the gel filtration time scale. It is likely that, for sub‐stoichiometric [2Fe‐2S]_2_‐WT‐anamorsin:[2Fe‐2S]^2+^_2_‐GLRX3_2_ ratios, a complex composed of homodimeric [2Fe‐2S]^2+^_2_‐GLRX3_2_ and one molecule only of [2Fe‐2S]_2_‐WT‐anamorsin is first formed and then, by further additions of [2Fe‐2S]_2_‐WT‐anamorsin, the hetero‐tetrameric complex is fully formed. Moreover, we observed that the weak peak of monomeric apo GLRX3 species (cyan line) disappears upon the further addition of [2Fe‐2S]_2_‐WT‐anamorsin, and concomitantly a new corresponding weak peak at 12.7 mL appears. The elution volume of the latter peak perfectly matches that of the apo GLRX3/[2Fe‐2S]_2_‐WT‐anamorsin heterodimeric complex (Figure 5C, purple line). These data indicates that the low amount of apo GLRX3 is complexed with [2Fe‐2S]_2_‐WT‐anamorsin when the latter protein is added in high amounts, in agreement with what previously observed by us for the apo GLRX3/[2Fe‐2S]_2_‐WT‐anamorsin complex formation (Banci, Ciofi‐Baffoni, et al., 2015). Analytical SEC was also performed on a 1:2 [2Fe‐2S]^2+^_2_‐GLRX3_2_/[2Fe‐2S]‐M1‐anamorsin mixture, to assess whether the deletion of the last 49 residues, which include the M2‐motif of anamorsin, affects the interaction with [2Fe‐2S]^2+^_2_‐GLRX3_2_. [2Fe‐2S]‐M1‐anamorsin alone elutes as a main single peak at 14.2 mL, with MW_app_ = 75.2 kDa that is higher than the theoretical molecular mass (29.7 kDa) (Table S1), similarly to what observed for the full‐length protein. The 1:2 [2Fe‐2S]^2+^_2_‐GLRX3_2_/[2Fe‐2S]‐M1‐anamorsin mixture elutes with a main peak at 11.6 mL and smaller peaks at 12.9 mL and 14.2 mL (Figure S7), corresponding, respectively, to an hetero‐tetrameric [2Fe‐2S]^2+^_2_‐GLRX3_2_/([2Fe‐2S]‐M1‐anamorsin)_2_ complex with MW_app_ = 277.3 kDa (theoretical MW_app_ = 282.9 kDa), to an apo GLRX3/[2Fe‐2S]‐M1‐anamorsin hetero‐dimeric 1:1 complex with MW_app_ = 129.3 kDa (theoretical MW_app_ = 137.6 kDa), and to [2Fe‐2S]‐M1‐anamorsin (Table S1), in agreement with the analysis of the protein content of the collected chromatographic fractions resulting from SDS‐PAGE (Figure S7).

Analytical SEC was also applied to investigate changes in the quaternary structure of the hetero‐tetrameric complex after its interaction with apo NUBP1. Specifically, the untagged hetero‐tetrameric complex was incubated with His_6_‐tagged apo NUBP1 and the products of the [4Fe‐4S] cluster assembly reaction were analyzed by SEC and by SDS‐PAGE before and after their separation by Ni^2+^‐affinity chromatography. The mixture of the hetero‐tetrameric complex and apo NUBP1 elutes as two peaks at 12.7 and 13.4 mL (Figure 5C), corresponding respectively to a GLRX3/anamorsin hetero‐complex and to His_6_‐tagged NUBP1, as showed by SDS‐PAGE (Figure S1) and by analytical SEC of the two isolated species, which elute with the same elution volumes once separated by Ni^2+^‐affinity chromatography (Figure 5C, purple and magenta lines, respectively). Interestingly, after the [4Fe‐4S] assembly reaction, the GLRX3/anamorsin complex elutes as a main single peak at 12.7 mL (Figure 5C). This elution volume is lower than that observed for the starting hetero‐tetrameric GLRX3‐anamorsin complex, which is thus not present anymore. The apparent mass of this new peak (MW_app_ = 142.7 kDa, Table S1) corresponds to that of the apo GLRX3/[2Fe‐2S]_2_‐WT‐anamorsin heterodimeric complex (Figure 5C, purple line). Accordingly, after separation, the Ni^2+^ column‐bound fraction containing His_6_‐tagged [4Fe‐4S]^2+^ NUBP1 elutes as a main single peak at 13.4 mL, which is the same elution volume found for apo NUBP1 (Figure 5C).

Overall, these results indicate that the two GLRX3 molecules, bridged by the two [2Fe‐2S]^2+^ clusters in the hetero‐tetrameric [2Fe‐2S]_2_‐GLRX3_2_/([2Fe‐2S]_2_‐anamorsin)_2_ complex, separate each other once the two [2Fe‐2S]^2+^ clusters are transferred to apo NUBP1, giving rise to the heterodimeric apo GLRX3/[2Fe‐2S]_2_‐WT‐anamorsin complex and [4Fe‐4S]^2+^ NUBP1 as final products of the process.

## 3 DISCUSSION

**SCHEME 1:**
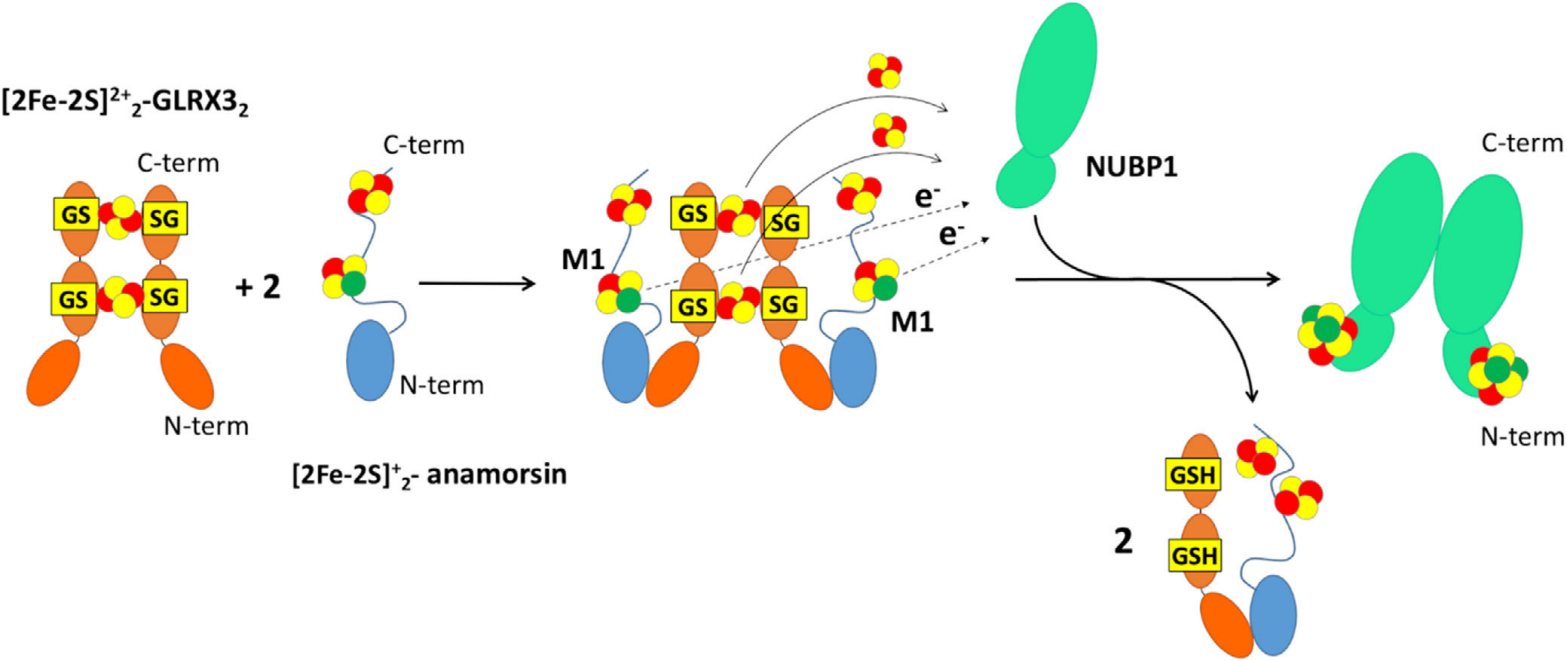
Proposed model for the [4Fe‐4S]^2+^ cluster assembly process on the N‐terminal site of NUBP1. [2Fe‐2S]^2+^_2_‐GLRX3_2_ and [2Fe‐2S]^+^_2_‐anamorsin interact forming a 1:2 [2Fe‐2S]^2+^_2_‐GLRX3_2_:[2Fe‐2S]^+^_2_‐anamorsin hetero‐tetrameric complex. The complex formation is driven by specific protein–protein recognition between the two N‐terminal domains of GLRX3 and anamorsin, according to what previously reported (Banci, Ciofi‐Baffoni, et al., 2015). The hetero‐tetrameric complex transiently interact with NUBP1 and transfers the two [2Fe‐2S]^2+^ clusters of GLRX3 and two electrons from the reduced M1‐bound [2Fe‐2S]^+^ clusters of anamorsin to assemble a [4Fe‐4S]^2+^ cluster on the N‐terminal site of NUBP1. As a result of both cluster and electron transfer, the hetero‐tetrameric complex is converted into a 1:1 apo GLRX3:[2Fe‐2S]_2_‐anamorsin complex. Fe–S clusters are represented as yellow spheres (sulfur atoms) and red spheres (Fe^3+^) or green spheres (Fe^2+^). GS stands for the cluster‐bound form of glutathione (GSH), which interacts with GLRX3 fulfilling the [2Fe‐2S] cluster first coordination sphere in [2Fe‐2S]^2+^_2_‐GLRX3_2_. After the cluster transfer event, GSH still interacts with GLRX3.

In this work, we unraveled the mechanism of [4Fe‐4S]^2+^ cluster assembly on the N‐terminal cluster binding site of NUBP1. We found that the two components of the CIA machinery GLRX3 and anamorsin are able to assemble the [4Fe‐4S]^2+^ cluster at the N‐terminal site of NUBP1. We showed that [2Fe‐2S]^+^‐anamorsin acts as electron donor in the assembly of the N‐terminal [4Fe‐4S]^2+^ cluster of NUBP1, while dimeric [2Fe‐2S]^2+^_2_‐GLRX3_2_ works as [2Fe‐2S]^2+^ cluster donor. Specifically, our study indicated that two [2Fe‐2S]^2+^ clusters, donated by [2Fe‐2S]^2+^_2_‐GLRX3_2_, are reductively coupled to form a [4Fe‐4S]^2+^ cluster at the N‐terminal site of NUBP1 and that the two needed electrons are supplied by the reduced [2Fe‐2S]^+^ cluster bound to the M1‐motif of anamorsin. We identified the key intermediate of this process, that is the hetero‐tetrameric complex formed by the dimeric [2Fe‐2S]^2+^_2_‐GLRX3_2_ and two [2Fe‐2S]_2_^+^‐anamorsin molecules. This hetero‐complex complies with all the molecular features required to assemble a [4Fe‐4S]^2+^ cluster, as it contains the [2Fe‐2S]^2+^ clusters in dimeric GLRX3 that are prone to be transferred, and the electrons of the reduced [2Fe‐2S]^+^ cluster of the M1‐motif of the two anamorsin molecules (Scheme 1). Upon the [4Fe‐4S]^2+^ cluster assembly on the N‐terminal site of NUBP1, the GLRX3/anamorsin hetero‐tetrameric complex partially splits up, forming an apo GLRX3/[2Fe‐2S]_2_‐anamorsin heterodimer as a consequence of the transfer of the two [2Fe‐2S]^2+^ clusters of [2Fe‐2S]_2_‐GLRX3_2_ to NUBP1 (Scheme 1). Indeed, the two [2Fe‐2S]^2+^ clusters of [2Fe‐2S]_2_‐GLRX3_2_ are the prerequisite to hold together the two GLRX3 molecules in the GLRX3/anamorsin hetero‐tetrameric complex (Haunhorst et al., 2010; Li et al., 2012).

Although our work does not address the specific sequence of events for the formation of the [4Fe‐4S]^2+^ cluster on NUBP1 involving the electron transfer between the M1‐bound [2Fe‐2S]^+^ cluster of anamorsin and the [2Fe‐2S]^2+^ clusters of [2Fe‐2S]_2_‐GLRX3_2_, we can make some relevant considerations on this aspect. In a previous study by us, we showed that the interaction between the N‐terminal domains of anamorsin and GLRX3 is essential for the formation of the complex between the two proteins, while the Fe–S cluster binding domains of [2Fe‐2S]_2_‐anamorsin and of [2Fe‐2S]_2_‐GLRX3_2_ are not involved in any permanent interaction (Banci, Ciofi‐Baffoni, et al., 2015). This finding agrees with what we find now by analytical SEC, that is, that the redox state of the clusters bound to anamorsin does not impact on complex formation between the two proteins. On the other hand, it is expected that, during the [4Fe‐4S] cluster assembly process, the cluster‐binding CIAPIN1 domain of anamorsin approaches transiently the N‐terminal cluster‐binding region of NUBP1, as required to donate two electrons for the reductive coupling of the two GLRX3‐donated [2Fe‐2S]^2+^ clusters into the [4Fe‐4S]^2+^ cluster. Indeed, transient interactions are typically observed in electron transfer processes (Prudêncio & Ubbink, 2004). The high structural flexibility of the linker and of the CIAPIN1 domain of anamorsin is well suited to allow the conformational changes required to drive the transient interaction between the reduced M1‐bound [2Fe‐2S]^+^ cluster of anamorsin and the [2Fe‐2S]^2+^ clusters of GLRX3 to reduce the latter clusters and thus to form the [4Fe‐4S]^2+^ cluster on NUBP1. We also showed that the reduced [2Fe‐2S]^+^ cluster bound to the M2‐motif of anamorsin does not have any role in the assembly of the [4Fe‐4S]^2+^ cluster on the N‐terminal site of NUBP1, and that the electron transfer from the [2Fe‐2S]^+^ cluster bound to the M1 site occurs independently of the presence of the [2Fe‐2S] cluster in the M2 site. This model is consistent with previous data showing that, in the physiological electron transfer chain composed of NDOR1 and anamorsin, the reduced FMN moiety of NDOR1 is able to transfer electrons exclusively to the cluster bound to the M1‐motif of anamorsin, and not to the cluster bound to the M2‐motif (Banci, Bertini, et al., 2013; Banci, Ciofi‐Baffoni, et al., 2013; Netz et al., 2010). In summary, these findings suggest a model where the [2Fe‐2S] cluster bound to the M2‐motif of anamorsin does not take part in the electron transfer chain required to assemble the N‐terminal [4Fe‐4S]^2+^ cluster of NUBP1. Our data also showed that the [2Fe‐2S] cluster bound to the M2‐motif of human anamorsin is not involved in cluster transfer to NUBP1. In vivo data are required to address the specific functional role of M2 motif of anamorsin. The role of this second Fe–S cluster has been only investigated in yeast, where the M2‐motif is essential for yeast cell viability and for cytosolic [4Fe‐4S] proteins activity (Netz et al., 2016; Zhang et al., 2017). However, this functional data on yeast cannot be applied to human anamorsin as M2‐motif of Dre2 binds a [4Fe‐4S] cluster while anamorsin binds a [2Fe‐2S] cluster, and this different cluster type can significantly differentiate the functional role of the M2‐motif in the two organisms.

The process that we have described in this work should be framed in a sequence of molecular events that take place in the cell, all contributing to define the early stages of the CIA machinery, where GLRX3 acts as a cluster chaperone for both [2Fe‐2S]‐ and [4Fe‐4S]‐cluster containing proteins. Initially, GLRX3 and anamorsin interact for the GLRX3‐mediated [2Fe‐2S]_2_‐anamorsin maturation process, where [2Fe‐2S]_2_‐GLRX3_2_ or GLRX3‐[2Fe‐2S]_2_‐BOLA2_2_ complexes transfer their [2Fe‐2S] clusters to apo anamorsin, upon specific interaction between the N‐terminal domain of GLRX3 and the N‐terminal domain of anamorsin (Banci, Camponeschi, et al., 2015; Banci, Ciofi‐Baffoni, et al., 2015; Frey et al., 2016). The mature, cluster‐bound form of anamorsin is then able to receive electrons from the reduced FMN moiety of NDOR1, thus enabling it to perform its function as an electron donor within the CIA machinery. After GLRX3‐driven anamorsin maturation, the next cellular step involves the interaction between two molecules of [2Fe‐2S]_2_‐anamorsin and one dimeric [2Fe‐2S]_2_‐GLRX3_2_ species to form the here identified key intermediate that provides two electrons (from the NDOR1‐reduced M1‐bound clusters of two anamorsin molecules) and two [2Fe‐2S]^2+^ clusters (from [2Fe‐2S]_2_‐GLRX3_2_) that are reductively coupled to form a [4Fe‐4S]^2+^ cluster at the N‐terminal site of NUBP1. The protein–protein recognition that involves the N‐terminal domains of GLRX3 and anamorsin can therefore drive two distinct cellular processes, that is, the maturation of anamorsin itself and that of NUBP1. In one case, the interaction between the N‐terminal domains can bring the two cysteine‐rich motives of anamorsin closer to the two [2Fe‐2S] clusters of [2Fe‐2S]_2_‐GLRX3_2_ or GLRX3‐[2Fe‐2S]_2_‐BOLA2_2_ thus promoting the transfer of the [2Fe‐2S] clusters to apo anamorsin. In the other case, the recognition between the two N‐terminal domains of GLRX3 and anamorsin drives the formation of a hetero‐tetrameric complex that is able to transfer and reductively couple the two [2Fe‐2S] clusters of GLRX3 to form a [4Fe‐4S] cluster at the N‐terminal site of NUBP1.

Our model supports a critical function of GLRX3 as [2Fe‐2S] cluster donor to assemble cytosolic [2Fe‐2S]‐ and [4Fe‐4S]‐cluster containing proteins, but this contrasts with the reported moderate deficiencies of cytosolic Fe–S cluster enzymes resulting from the depletion of GLRX3 in mammalian cells (Haunhorst et al., 2010). The most reasonable explanation for the relatively weak in vivo phenotype of GLRX3 is that GLRX3‐dependent pathways described above by us can be likely bypassed in the cells via the activation of alternative pathways, that is, redundant systems can partially substitute for GLRX3 function in mammalian cells. This effect has been already proposed in the literature when the role of GLRX3 in providing Fe–S cluster to anamorsin was studied (Frey et al., 2016). It was indeed reported that anamorsin may be capable of acquiring Fe–S clusters from a source alternative to GLRX3, for example, the NEET family of [2Fe‐2S] proteins (Tamir et al., 2015). Thus, it is possible that the same or alternative systems are activated for the formation of the N‐terminal [4Fe‐4S] cluster of NUBP1. A model where mitoNEET or miner1 substitute GLRX3 as [2Fe‐2S] cluster donors, although still requiring experimental evidences, is possible for two reasons: 1. both mitoNEET and miner1 have molecular features similar to those of homodimeric GLRX3, which are those required to form a [4Fe‐4S] cluster on NUBP1, that is, they have two easily transferable [2Fe‐2S] clusters bound in a homodimer, as previously shown (Lipper et al., 2015); 2. both [2Fe‐2S] clusters of mitoNEET are reduced by a complex composed by [2Fe‐2S]‐anamorsin and the FMN binding domain of NDOR1 via the formation of a transient complex that brings the [2Fe‐2S]^2+^ clusters of mitoNEET close to the reduced [2Fe‐2S]^+^ cluster bound to M1‐motif of anamorsin (Camponeschi et al., 2017).

## 4 CONCLUSIONS

This work contributes to the molecular understanding of the mechanism of [4Fe‐4S] proteins biogenesis in the cytosol, showing that anamorsin and GLRX3 act as an electron donor and as a [2Fe‐2S] cluster donor, respectively, in the assembly of the N‐terminal [4Fe‐4S] cluster of NUBP1. Our studies allowed us to propose that the dimeric [2Fe‐2S]^2+^_2_‐GLRX3_2_ complex and two molecules of [2Fe‐2S]_2_^+^‐anamorsin form a hetero‐tetrameric complex that acts as a component of the CIA machinery at its early stages. The hetero‐tetrameric complex is able to provide two [2Fe‐2S]^2+^ clusters from the GLRX3 dimeric unit and two electrons, one from each reduced [2Fe‐2S]^+^ cluster bound to the M1‐motif of anamorsin, to assemble a [4Fe‐4S]^2+^ cluster on the N‐terminal site of NUBP1. On the contrary, the [2Fe‐2S] cluster bound to the M2‐motif of anamorsin is not involved in any step of this [4Fe‐4S] cluster assembly process.

## 5 MATERIALS AND METHODS

### 5.1 Protein production

Apo full‐length wild‐type NUBP1, NUBP1_NT_, NUBP1_CT_, homodimeric [2Fe‐2S]_2_‐GLRX3_2_ and [2Fe‐2S]^2+^ WT‐ M1‐ and M2‐anamorsin proteins were expressed and purified following previously reported procedures (Banci, Ciofi‐Baffoni, et al., 2015; Camponeschi et al., 2020; Matteucci et al., 2021).

Reduced [2Fe‐2S]_2_^+^‐WT‐, [2Fe‐2S]^+^‐M1‐ and ‐M2‐anamorsin were obtained by adding stoichiometric amounts of sodium dithionite to fully reduce all clusters. The buffer was then exchanged by PD10 desalting column (Cytiva) to remove any excess of sodium dithionite. The reduction of anamorsin clusters was monitored by UV–vis and EPR spectroscopies (Figure S8).

### 5.2 Protein, iron and acid‐labile sulfide quantification

Protein quantification was carried out with the Bradford protein assay, using BSA as a standard. Non‐heme iron and acid‐labile sulfide content was determined as described previously (Banci et al., 2011).

### 5.3 Biochemical and spectroscopic UV–vis, NMR and EPR methods

The quaternary structure of the proteins was analyzed through analytical SEC on a Superdex 200 10/300 Increase column (Cytiva). The column was calibrated with gel filtration marker calibration kit, 12.4–2000 kDa (Sigma–Aldrich), to obtain the apparent molecular masses of the detected species. Samples were loaded on the pre‐equilibrated column with degassed 50 mM sodium phosphate buffer pH 8.0, NaCl 200 mM, 5 mM GSH. Elution profiles were recorded at 280 nm with a flow rate of 0.7 mL/min. Protein concentration was in the 0.1–0.2 mM range.

UV–vis spectra were anaerobically acquired on a Cary 50 Eclipse spectrophotometer.

Paramagnetic 1D ^1^H NMR experiment was performed on a Bruker Avance spectrometer operating at 400 MHz ^1^H Larmor frequency an equipped with a ^1^H optimized 5 mm probe. Water signal was suppressed via fast repetition experiments and water selective irradiation (Patt & Sykes, 1972). Experiments were typically performed using an acquisition time of 50 ms, and an overall recycle delay of 80 ms. Sample concentration was in the range 0.2–0.4 mM, in degassed 50 mM Tris buffer pH 8.0, 150 mM NaCl, 100% D_2_O. Squared cosine and exponential multiplications were applied prior to Fourier Transformation. Manual baseline correction was performed using polynomial functions.

CW EPR spectra were recorded after the anaerobic reduction of the protein by addition of 1 eq. of sodium dithionite, before and after buffer exchanging the protein with PD10 desalting column. Protein concentration was in the 0.1–0.2 mM range, in degassed 50 mM Tris buffer pH 8.0, 150 mM NaCl, and 10% glycerol or 50 mM sodium phosphate buffer pH 8.0, 200 mM NaCl, and 10% glycerol. EPR spectra were acquired at 10 K and 45 K, using a Bruker Elexsys 580 spectrometer working at a microwave frequency of ca. 9.36 GHz, equipped with a SHQ cavity and a continuous flow He cryostat (ESR900, Oxford instruments) for temperature control. Acquisition parameters were as follows: microwave frequency, 9.36 GHz; microwave power, 1 mW at 10 K, 0.12 mW at 45 K; modulation frequency, 100 kHz; modulation amplitude, 10 G; acquisition time constant, 163.84 ms; number of points 1024; number of scans 4; field range 2000–4000 G.

### 5.4 Cluster transfer from [2Fe‐2S] _2_‐GLRX3_2_ /[2Fe‐2S]^+^‐anamorsin to His_6_‐tagged apo NUBP1

His_6_‐tagged apo NUBP1 was incubated under anaerobic conditions with 1.5 eq. of homodimeric [2Fe‐2S]^2+^_2_‐GLRX3_2_, and 3.0 eq. of monomeric reduced, [2Fe‐2S]^+^_2_‐WT‐anamorsin or 3.0 eq. of monomeric reduced, [2Fe‐2S]^+^‐M1‐anamorsin or [2Fe‐2S]^+^‐M2‐anamorsin for 1 hour at room temperature in 50 mM sodium phosphate buffer pH 8.0, 200 mM NaCl and 5 mM imidazole. The final His_6_‐tagged apo NUBP1:[2Fe‐2S]_2_‐GLRX3_2_:[2Fe‐2S]^+^‐anamorsin ratio correspond to the stoichiometric amounts of [2Fe‐2S] clusters from [2Fe‐2S]_2_‐GLRX3_2_ and electrons from [2Fe‐2S] ^+^‐anamorsin, required to fully saturate the cluster binding sites present in NUBP1 with a [4Fe‐4S]^2+^ cluster, that is, three per dimeric NUBP1 (Camponeschi et al., 2020). Separation of His_6_‐tagged NUBP1 from untagged GLRX3 and untagged anamorsin after the reaction was performed in anaerobic conditions, by loading the reaction mixtures on a His GraviTrap column pre‐equilibrated with 50 mM sodium phosphate buffer pH 8.0, 200 mM NaCl and 5 mM imidazole. The His_6_‐tagged NUBP1 species was eluted with 50 mM sodium phosphate buffer pH 8.0, 200 mM NaCl and 400 mM imidazole. After concentration the buffer was exchanged by PD‐10 desalting column in the appropriate degassed buffer required to perform analytical gel filtration, iron and acid‐labile sulfide quantification and to acquire UV–vis and paramagnetic 1D ^1^H NMR spectra.

## AUTHOR CONTRIBUTIONS

**Beatrice Bargagna:** Investigation (equal); validation (equal); writing – review and editing (equal). **Sara Matteucci:** Investigation (equal); validation (equal); writing – review and editing (equal). **Simone Ciofi‐Baffoni:** Conceptualization (equal); writing – original draft (equal); writing – review and editing (equal). **Francesca Camponeschi:** Conceptualization (equal); investigation (equal); validation (equal); visualization (lead); writing – original draft (equal); writing – review and editing (equal). **Lucia Banci:** Conceptualization (equal); funding acquisition (lead); project administration (lead); supervision (lead); writing – review and editing (equal).

## CONFLICT OF INTEREST STATEMENT

The authors declare no conflict of interest.

## Supporting information


**Data S1.** Eight figures reporting: SDS‐PAGE proteins content analysis of the collected fraction after Ni‐affinity chromatography on the NUBP1/[2Fe‐2S]_2_‐GLRX3_2_ and [2Fe‐2S]‐WT‐, M1‐ or M2‐anamorsin reaction mixtures; the temperature dependence of the paramagnetic 1D ^1^H NMR signals of HisNUBP1 after mixing with [2Fe‐2S]_2_‐GLRX3_2_ and [2Fe‐2S]_2_‐WT‐anamorsin; UV–vis spectra of His_6_‐tagged NUBP1_NT_ and His_6_‐tagged NUBP1_CT_ before and after the incubation and isolation from [2Fe‐2S]^2+^_2_‐GLRX3_2_ and [2Fe‐2S]^+^_2_‐anamorsin; UV–vis spectra of full‐length wild‐type His_6_‐tagged NUBP1 isolated after the incubation with [2Fe‐2S]^+^_2_‐anamorsin in the absence of [2Fe‐2S]^2+^_2_‐GLRX3_2_; UV–vis spectra of His_6_‐tagged NUBP1 isolated after the incubation with [2Fe‐2S]^2+^_2_‐GLRX3_2_ in the absence of anamorsin and in the presence of cluster‐oxidized [2Fe‐2S]^2+^_2_‐anamorsin; CD spectra of His_6_‐tagged NUBP1 isolated after the incubation with [2Fe‐2S]^2+^_2_‐GLRX3_2_ and cluster‐reduced [2Fe‐2S]^+^‐M2‐anamorsin; SEC and SDS‐PAGE analysis of the protein content of the collected chromatographic fractions of [2Fe‐2S]^2+^_2_‐GLRX3_2_/[2Fe‐2S]‐M1‐anamorsin 1:2 mixture; UV–vis and EPR spectra of cluster‐reduced WT‐anamorsin. One table reporting the theoretical and apparent molecular masses of [2Fe‐2S]^2+^_2_‐GLRX3_2_ and [2Fe‐2S]^+^‐anamorsin proteins and of their complexes, as estimated by size exclusion chromatography.

## Data Availability

The data that support the findings of this study are available from the corresponding author upon reasonable request.
