# Supplementary material for: Unraveling the mechanism of [4Fe‐4S] cluster assembly on the N‐terminal cluster binding site of NUBP1
Source: Protein Sci. 2023 May 1;32(5):e4625. doi: 10.1002/pro.4625 (PMC10108433; doi:10.1002/pro.4625)
Supplement: Supplementary file 1 — Data S1. Eight figures reporting: SDS‐PAGE proteins content analysis of the collected fraction after Ni‐affinity chromatography on the NUBP1/[2Fe‐2S]2‐GLRX32 and [2Fe‐2S]‐WT‐, M1‐ or M2‐anamorsin reaction mixtures; the temperature dependence of the paramagnetic 1D 1H NMR signals of HisNUBP1 after mixing with [2Fe‐2S]2‐GLRX32 and [2Fe‐2S]2‐WT‐anamorsin; UV–vis spectra of His6‐tagged NUBP1NT and His6‐tagged NUBP1CT before and after the incubation and isolation from [2Fe‐2S]2+ 2‐GLRX32 and [2Fe‐2S]+ 2‐anamorsin; UV–vis spectra of full‐length wild‐type His6‐tagged NUBP1 isolated after the incubation with [2Fe‐2S]+ 2‐anamorsin in the absence of [2Fe‐2S]2+ 2‐GLRX32; UV–vis spectra of His6‐tagged NUBP1 isolated after the incubation with [2Fe‐2S]2+ 2‐GLRX32 in the absence of anamorsin and in the presence of cluster‐oxidized [2Fe‐2S]2+ 2‐anamorsin; CD spectra of His6‐tagged NUBP1 isolated after the incubation with [2Fe‐2S]2+ 2‐GLRX32 and cluster‐reduced [2Fe‐2S]+‐M2‐anamorsin; SEC and SDS‐PAGE analysis of the protein content of the collected chromatographic fractions of [2Fe‐2S]2+ 2‐GLRX32/[2Fe‐2S]‐M1‐anamorsin 1:2 mixture; UV–vis and EPR spectra of cluster‐reduced WT‐anamorsin. One table reporting the theoretical and apparent molecular masses of [2Fe‐2S]2+ 2‐GLRX32 and [2Fe‐2S]+‐anamorsin proteins and of their complexes, as estimated by size exclusion chromatography. [file PRO-32-e4625-s001.docx]

**Supporting Information**

**for**

**Unraveling the mechanism of [4Fe-4S] cluster assembly on NUBP1**

Beatrice Bargagna^1,§^, Sara Matteucci^1,§^, Simone Ciofi-Baffoni^1,2,3^, Francesca Camponeschi^1,2,3,*^ Lucia Banci^1,2,3,*^

*^1^ Magnetic Resonance Center CERM, University of Florence, Via L. Sacconi 6, 50019 Sesto Fiorentino, Florence (Italy)*

*^2^ Consorzio Interuniversitario Risonanze Magnetiche di Metalloproteine (CIRMMP)Via L. Sacconi 6, 50019 Sesto Fiorentino, Florence (Italy)*

*^3^ Department of Chemistry, University of Florence Via della Lastruccia 3, 50019 Sesto Fiorentino, Florence (Italy)*

** banci@cerm.unifi.it, camponeschi@cerm.unifi.it*

^§^ Equal contribution

**CONTENTS**

**Figure S1.** SDS-PAGE analysis of the protein content of the fractions collected after incubation and separation of [2Fe-2S]^2+^_2_-GLRX3_2_, [2Fe-2S]^+^_2_-WT-anamorsin, [2Fe-2S]^+^-M1-anamorsin, [2Fe-2S]^+^-M2-anamorsin and apo full-length wild-type His_6_-tagged NUBP1.

**Figure S2.** Temperature dependence of the paramagnetic 1D ^1^H NMR signals of NUBP1 after incubation and isolation from [2Fe-2S]^2+^_2_-GLRX3_2_ and full-length wild-type [2Fe-2S]^+^_2_- anamorsin.

**Figure S3.** UV-vis spectra of His_6_-tagged NUBP1_NT_ and His_6_-tagged NUBP1_CT_ before and after the incubation and isolation from [2Fe-2S]^2+^_2_-GLRX3_2_ and [2Fe-2S]^+^_2_-anamorsin.

**Figure S4.** UV-vis spectra of full-length wild-type His_6_-tagged NUBP1 before and after the incubation with [2Fe-2S]^+^_2_-anamorsin in the absence of [2Fe-2S]^2+^_2_-GLRX3_2_.

**Figure S5.** UV-vis spectra of His_6_-tagged NUBP1 isolated after the incubation with [2Fe-2S]^2+^_2_-GLRX3_2_ in the absence of anamorsin and in the presence of cluster-oxidized [2Fe-2S]^2+^_2_-anamorsin.

**Figure S6.** CD spectra of His_6_-tagged NUBP1 isolated after the incubation with [2Fe-2S]^2+^_2_-GLRX3_2_ and cluster-reduced [2Fe-2S]^+^_2_-WT-anamorsin, [2Fe-2S]^+^-M1-anamorsin [2Fe-2S]^+^-M2- anamorsin.

Figure S7. SEC and SDS-PAGE analysis of the protein content of the collected chromatographic fractions of [2Fe-2S]^2+^_2_-GLRX3_2_/[2Fe-2S]-M1-anamorsin 1:2 mixture.

**Figure S8.** UV-visible and EPR spectra of cluster reduced [2Fe-2S]^+^_2_-WT-anamorsin.

**Table S1.** Theoretical and apparent molecular masses of [2Fe-2S]^2+^_2_-GLRX3_2_ and [2Fe-2S]^+^-anamorsin proteins and of their complexes, as estimated by size exclusion chromatography.

**Supplementary Figures**

**
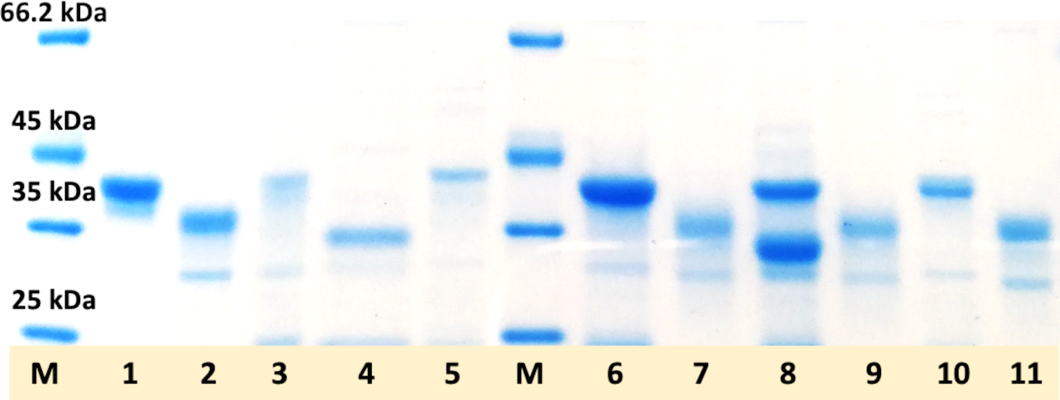
**

**Figure S1. SDS-PAGE of GLRX3, NUBP1, WT-, M1- and M2-anamorsin before incubation and after separation by Ni^2+^-affinity chromatography.** M = marker; Lanes 1 to 5 correspond, respectively, to isolated [2Fe-2S]_2_-GLRX3_2_, His_6_-tagged NUBP1, [2Fe-2S]_2_-WT-anamorsin, [2Fe-2S]-M1-anamorsin and [2Fe-2S]-M2-anamorsin before incubation; Lane 6 = GLRX3/[2Fe-2S]_2_-WT-anamorsin mixture after incubation and separation from His_6_-tagged NUBP1; Lane 7 = His_6_-tagged NUBP1 after incubation and separation from [2Fe-2S]^2+^_2_-GLRX3_2_/[2Fe-2S]^+^_2_-WT-anamorsin mixture; Lane 8 = GLRX3/[2Fe-2S]-M1-anamorsin mixture after incubation and separation from His_6_-tagged NUBP1; Lane 9 = His_6_-tagged NUBP1 after incubation and separation from [2Fe-2S]^2+^_2_-GLRX3_2_/[2Fe-2S]^+^-M1-anamorsin mixture; Lane 10 = GLRX3/[2Fe-2S]-M2-anamorsin mixture after incubation and separation from His_6_-tagged NUBP1; Lane 11 = His_6_-tagged NUBP1 after incubation and separation from [2Fe-2S]^2+^_2_-GLRX3_2_/[2Fe-2S]^+^-M2-anamorsin mixture.


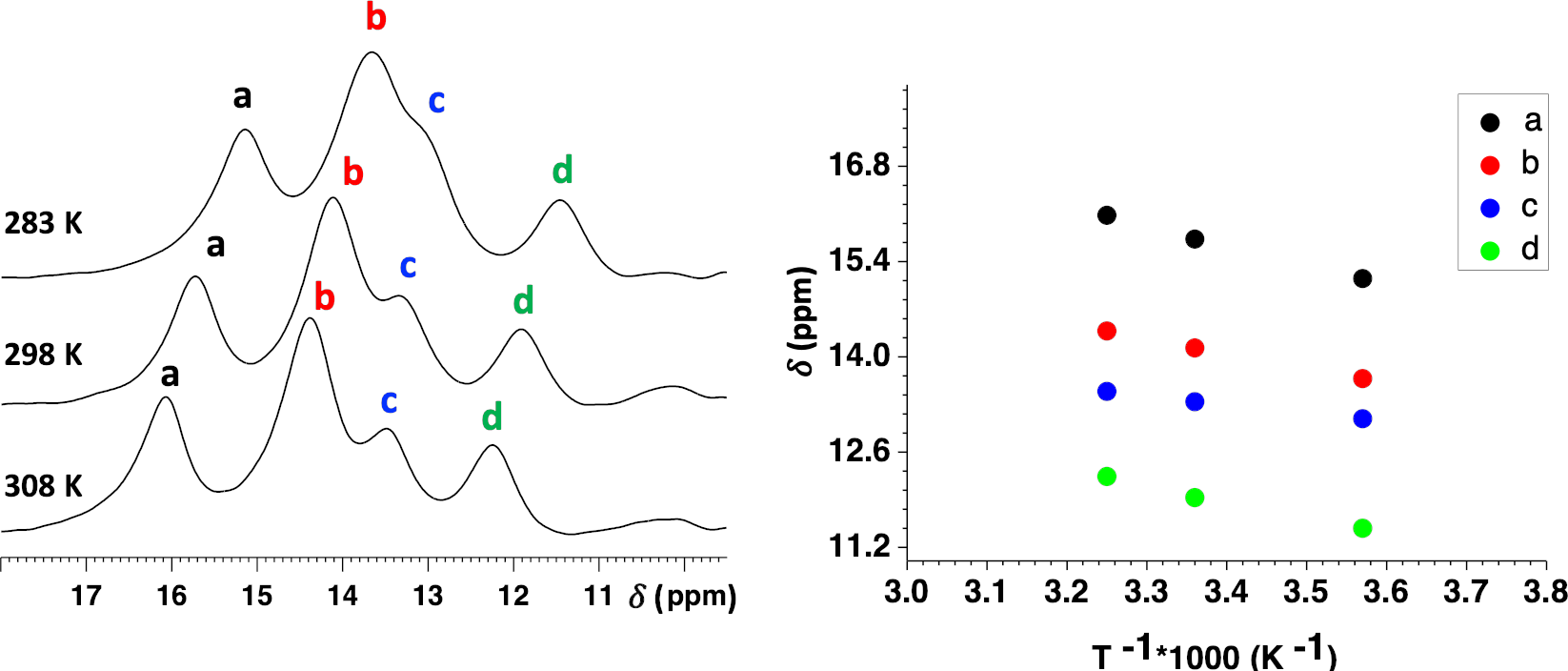


**Figure S2. Temperature dependence of the paramagnetic 1D ^1^H NMR signals of NUBP1 after incubation and isolation from [2Fe-2S]^2+^_2_-GLRX3_2_ and [2Fe-2S]^+^_2_-WT-anamorsin.**


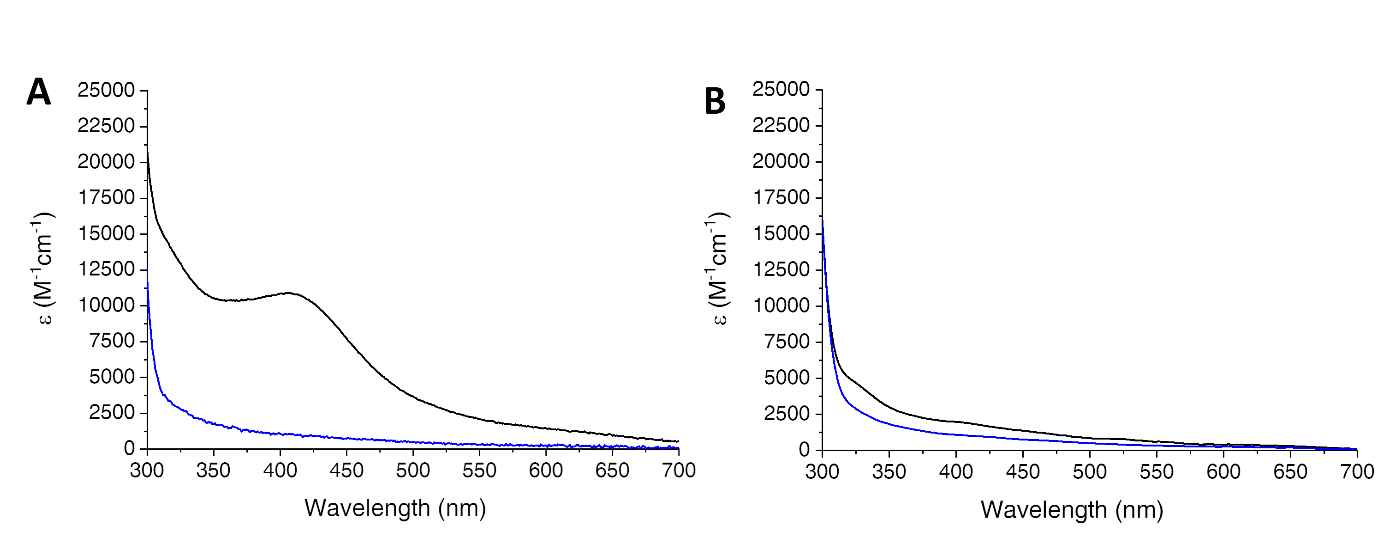


**Figure S3. [2Fe-2S]^2+^_2_-GLRX3_2_/[2Fe-2S]^+^_2_-anamorsin mixture promotes the assembly of a [4Fe-4S]^2+^ cluster at the N-terminal site of His_6_-tagged NUBP1 but not at the C-terminal site.** UV-vis spectra of A) His_6_-tagged NUBP1_NT_ and B) His_6_-tagged NUBP1_CT_ before (blue line) and after (black line) the incubation and isolation from [2Fe-2S]^2+^_2_-GLRX3_2_ and [2Fe-2S]^+^_2_-anamorsin.


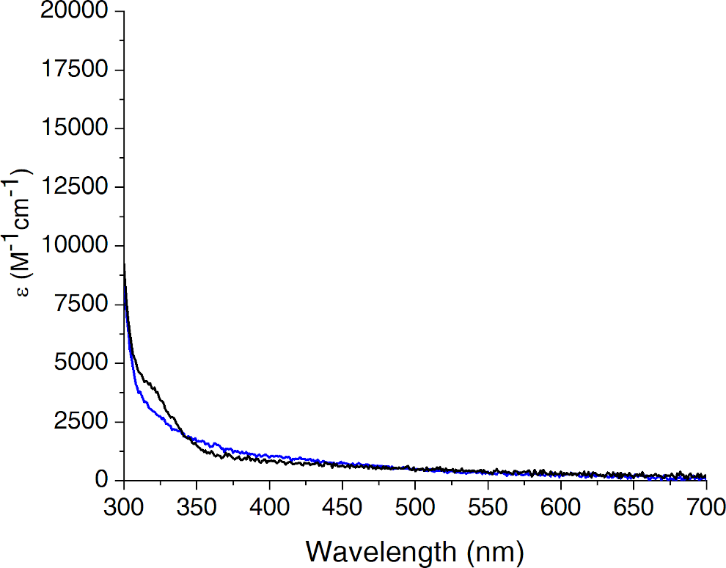


**Figure S4. [2Fe-2S]^2+^_2_-GLRX3_2_ is required for the transfer of Fe-S cluster to His_6_-tagged NUBP1.** UV-vis spectra of His_6_-tagged NUBP1 before (blue line) and after (black line) the incubation and isolation from cluster-reduced [2Fe-2S]^+^_2_-anamorsin in the absence of [2Fe-2S]^2+^_2_-GLRX3_2_.


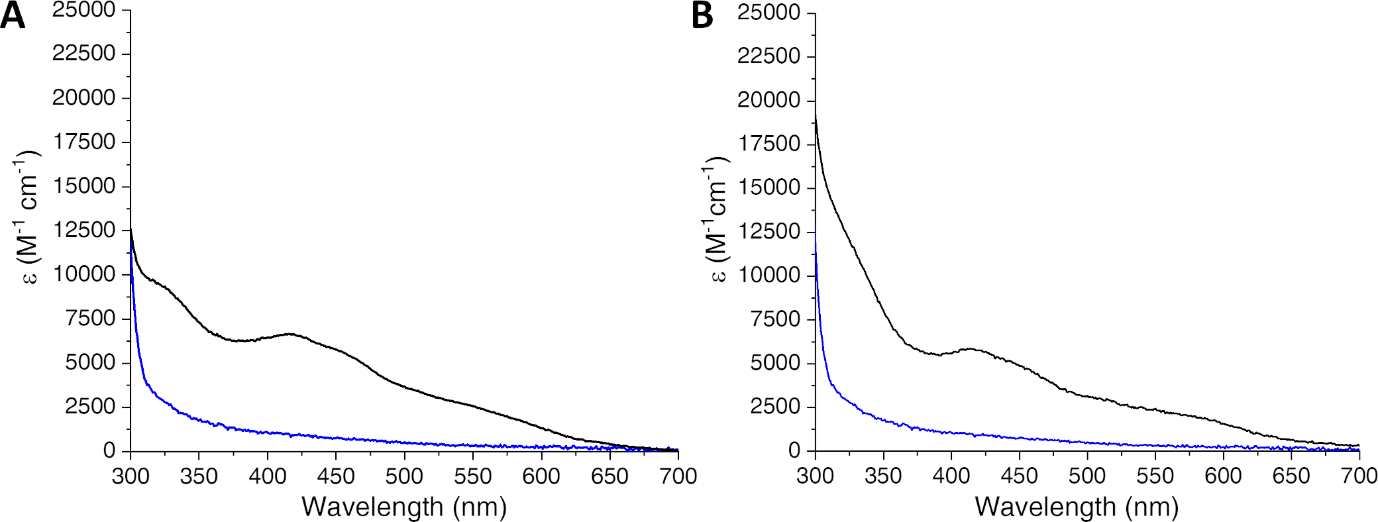


**Figure S5. [2Fe-2S]^+^_2_-anamorsin is required for the reductive coupling of two GLRX3-donated [2Fe-2S]^2+^ clusters on His_6_-tagged NUBP1.** UV-vis spectra of His_6_-tagged NUBP1 before (blue line) and after (black line) the incubation and isolation from [2Fe-2S]^2+^_2_-GLRX3_2_ in the absence of [2Fe-2S]^+^_2_-anamorsin (A) and in the presence of cluster-oxidized [2Fe-2S]^2+^_2_-anamorsin (B).


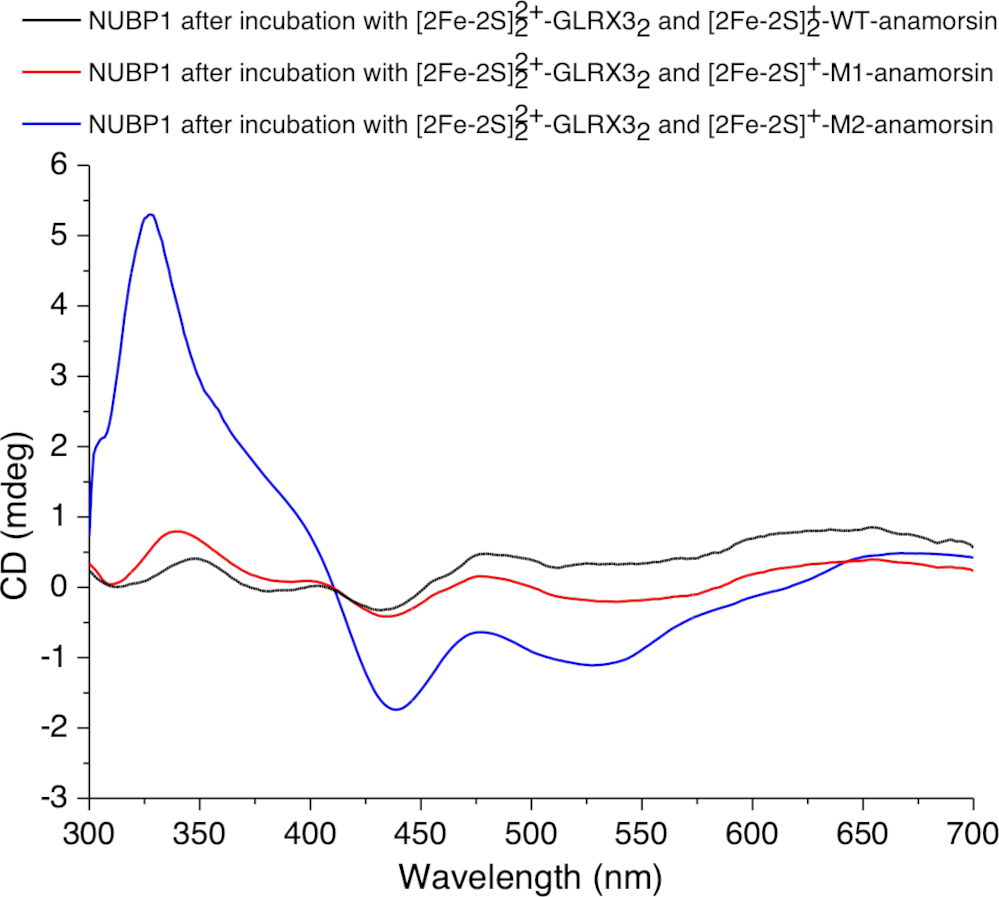


**Figure S6. CD spectra of His_6_-tagged NUBP1 after the incubation and isolation from [2Fe-2S]^2+^_2_-GLRX3_2_ and i) cluster-reduced [2Fe-2S]^+^_2_-WT-anamorsin (black line), ii) cluster-reduced [2Fe-2S]^+^-M1-anamorsin (red line) and iii) [2Fe-2S]^+^-M2-anamorsin (blue line).**


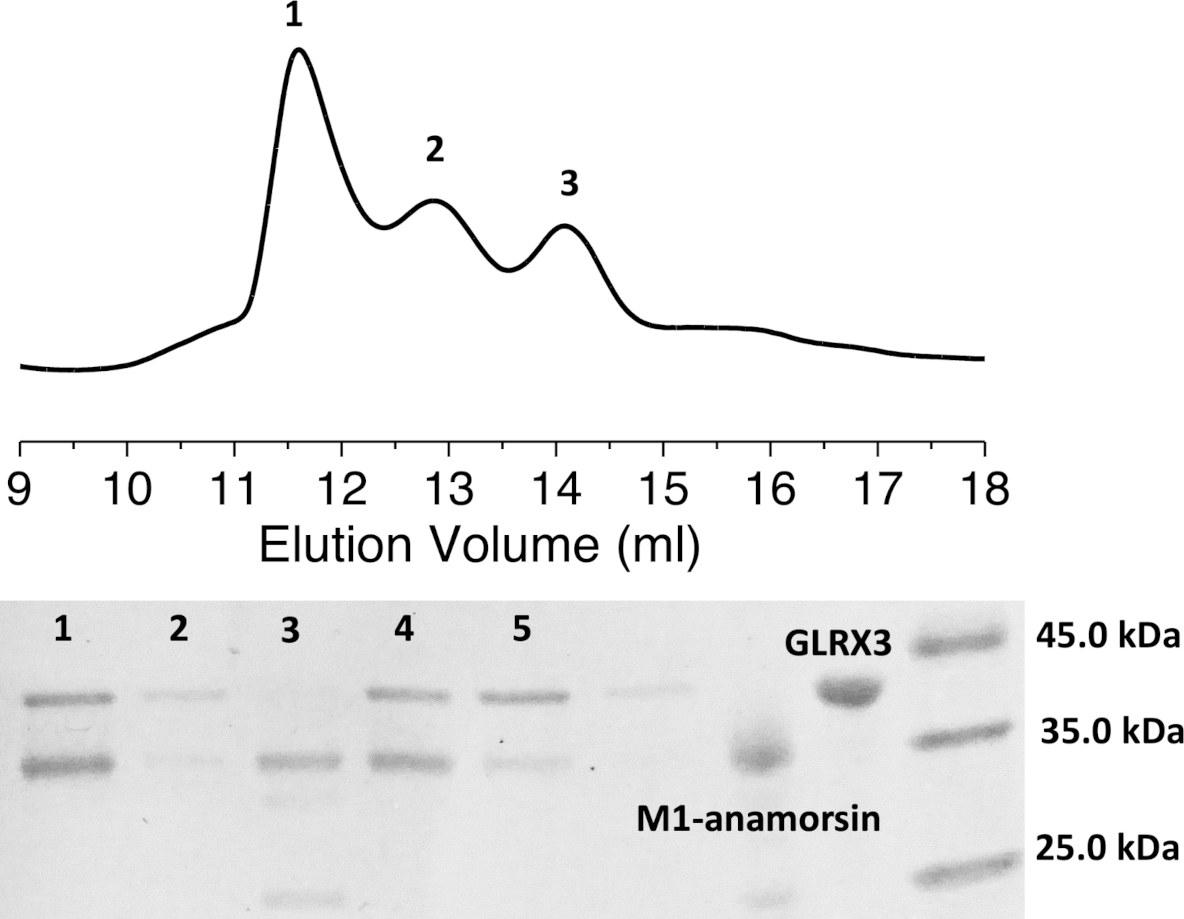


**Figure S7**. (A) SEC and (C) SDS-PAGE analysis of the protein content of the collected chromatographic fractions of [2Fe-2S]_2_-GLRX3_2_/[2Fe-2S]-M1-anamorsin 1:2 mixture. Lanes 1-3 correspond to peaks 1-3 in the gel filtration chromatogram; lane 4 correspond to a 1:2 dilution of sample reported in lane 1; lane 5 correspond to a 2X concentration of sample reported in lane 2.

**
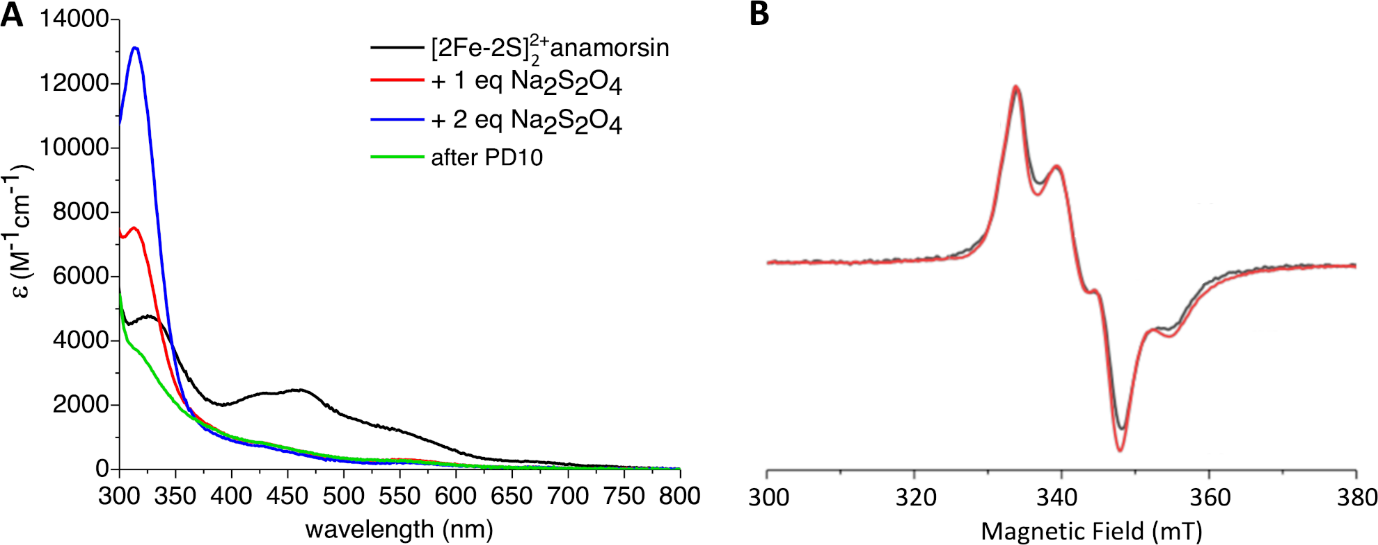
**

**Figure S8: UV-visible and EPR spectra of [2Fe-2S]_2_-anamorsin.** A) UV-vis spectra of [2Fe-2S]_2_-anamorsin before (black line) and after the reduction with 1 eq. (red line) and 2 eq. (blue line) of sodium dithionite. The protein remains in the reduced [2Fe-2S]^+^_2_-anamorsin form after the removal of the excess of dithionite by PD10 desalting column (green line). B) CW X-band EPR spectra of anaerobically purified [2Fe-2S]_2_-WT-anamorsin after reduction with 1 eq. of sodium dithionite at 45 K, before (red line) and after (black line) removing the excess of sodium dithionite by PD10 desalting column.

**Table S1. Theoretical and apparent molecular masses of GLRX3 and anamorsin proteins and protein-protein complexes, as estimated by analytical size exclusion chromatography.**

| **Species** | **Elution Volume (ml)** | **Apparent mass (kDa)^a^** | **Theoretical apparent mass (kDa)^b^** | **Theoretical mass (kDa)** |
| --- | --- | --- | --- | --- |
| apo GLRX3 | 14.60 | 62.4 | 62.4 | 37.8 |
| [2Fe-2S]_2_-GLRX3_2_ | 12.95 | 132.5 | 126.4 | 77.2 |
| [2Fe-2S]_2_-WT-anamorsin | 14.09 | 78.7 | 78.7 | 34.3 |
| [2Fe-2S]-M1-anamorsin | 14.19 | 75.2 | 75.2 | 29.7 |
| GLRX3:[2Fe-2S]_2_-WT-anamorsin  1:1 complex | 12.75 | 142.7 | 141.1 | 72.1 |
| [2Fe-2S]_2_-GLRX3_2_:[2Fe-2S]_2_-WT-anamorsin  1:2 complex | 11.23 | 288.4 | 289.8 | 145.9 |
| [2Fe-2S]_2_-GLRX3_2_:[2Fe-2S]_2_-WT-anamorsin  1:1 complex | 12.10 | 197.7 | 211.2 | 111.6 |
| GLRX3:[2Fe-2S]-M1-anamorsin  1:1 complex | 12.89 | 129.3 | 137.6 | 67.5 |
| [2Fe-2S]_2_-GLRX3_2_:[2Fe-2S]-M1-anamorsin  1:2 complex | 11.65 | 277.3 | 282.9 | 136.6 |

^a^ Estimated through analytical SEC

^b^ Calculated as the sum of the apparent masses estimated through analytical SEC
